# Preservation stress resistance of melanin deficient conidia from *Paecilomyces variotii* and *Penicillium roqueforti* mutants generated via CRISPR/Cas9 genome editing

**DOI:** 10.1186/s40694-021-00111-w

**Published:** 2021-04-02

**Authors:** Sjoerd J. Seekles, Pepijn P. P. Teunisse, Maarten Punt, Tom van den Brule, Jan Dijksterhuis, Jos Houbraken, Han A. B. Wösten, Arthur F. J. Ram

**Affiliations:** 1 TIFN, Agro Business Park 82, 6708 PW Wageningen, The Netherlands; 2 Department Molecular Microbiology and Biotechnology, Institute of Biology, Leiden University, Sylviusweg 72, 2333BE Leiden, The Netherlands; 3 Microbiology, Department of Biology, Utrecht University, Padualaan 8, 3584 CH Utrecht, The Netherlands; 4 Applied & Industrial Mycology, Westerdijk Fungal Biodiversity Institute, Uppsalalaan 8, 3584 CT Utrecht, the Netherlands

**Keywords:** CRISPR/Cas9, Cell factory, Melanin, Food spoilage, Food spoiling fungi, Polyketide synthase, Conidia, *Aspergillus niger*, *Penicillium roqueforti*, *Paecilomyces variotii*

## Abstract

**Background:**

The filamentous fungi *Paecilomyces variotii* and *Penicillium roqueforti* are prevalent food spoilers and are of interest as potential future cell factories. A functional CRISPR/Cas9 genome editing system would be beneficial for biotechnological advances as well as future (genetic) research in *P. variotii* and *P. roqueforti*.

**Results:**

Here we describe the successful implementation of an efficient AMA1-based CRISPR/Cas9 genome editing system developed for *Aspergillus niger* in *P. variotii* and *P. roqueforti* in order to create melanin deficient strains*.* Additionally, *kusA*^*−*^ mutant strains with a disrupted non-homologous end-joining repair mechanism were created to further optimize and facilitate efficient genome editing in these species. The effect of melanin on the resistance of conidia against the food preservation stressors heat and UV-C radiation was assessed by comparing wild-type and melanin deficient mutant conidia.

**Conclusions:**

Our findings show the successful use of CRISPR/Cas9 genome editing and its high efficiency in *P. variotii* and *P. roqueforti* in both wild-type strains as well as *kusA*^*−*^ mutant background strains. Additionally, we observed that melanin deficient conidia of three food spoiling fungi were not altered in their heat resistance. However, melanin deficient conidia had increased sensitivity towards UV-C radiation.

**Supplementary Information:**

The online version contains supplementary material available at 10.1186/s40694-021-00111-w.

## Introduction

The genome editing system by clustered regularly interspaced short palindromic repeats (CRISPR) and CRISPR-associated protein 9 (Cas9) has proven to be a powerful tool in filamentous fungi, providing new insights and opportunities within food, agricultural, clinical and biotechnological research [1–4]. Currently, the CRISPR/Cas9 gene editing tool has been introduced in over 40 species of filamentous fungi and oomycetes to date [5]. In this paper, we describe a functional CRISPR/Cas9 genome editing protocol for two food spoilage fungi *Paecilomyces variotii* and *Penicillium roqueforti*.

The CRISPR/Cas9 genome editing system introduces a double stranded break (DSB) on a specific genomic DNA site. Fungi have two main DNA repair mechanisms that can restore the DSB created by CRISPR/Cas9, namely the non-homologous end-joining repair mechanism (NHEJ) and the homology directed repair mechanism (HDR). For genome editing purposes, many studies rely on the HDR mechanism in order to control genomic editing (e.g. gene replacement studies), by providing the fungus with homologous DNA created in vitro [6]*.* This allows for precise DNA insertion, replacement or removal in the genome. However, many filamentous fungi prefer repair via NHEJ over HDR, which complicates this precise genome editing. In order to promote DNA repair by HDR in fungi, genes involved in the NHEJ repair mechanism can be deleted. A mutant fungus with a deleted *kusA* gene is defective in the NHEJ repair mechanism, therefore a DSB can only be repaired by HDR as shown in several filamentous fungi such including e.g. *Neurospora crassa* [7], *Aspergillus niger* [8].

The thermotolerant nature of *P. variotii* spores makes this fungus a relevant food spoiler [9–12]. *P. variotii* is a known spoiler of fruit juices, sauce, canned products and non-carbonized sodas [13, 14]. Additionally, *P. variotii* strains have been reported to produce industrially interesting, often thermostable, enzymes such as tannases, amylases, β-glucosidases and an alcohol oxidase [15–21]. Recently, a genome of *P. variotii* has been published [22] in which the first method on targeted gene disruptions in this fungus using *Agrobacterium tumefaciens* is described. Although *A. tumefaciens* mediated transformations are shown to be efficient and beneficial over other transformation methods in certain cases [23], it does require optimization of multiple factors and can be tedious compared to the relatively quick and easy to use PEG-mediated transformations [24].

The filamentous fungus *P. roqueforti* is best known as the ‘blue cheese’ fungus for its use in blue cheese production [25]. However, *P. roqueforti* is also a known food spoiler that can produce mycotoxins such as PR-toxin and roquefortine-C, which form potential health risks for humans [26–29]. As such, *P. roqueforti* has been intensively studied for its secondary metabolite production and specifically its mycotoxin production [30–33]. Additionally, *P. roqueforti* has biotechnological potential as a cell factory, as it produces proteolytic enzymes of interest to the cheese-making industry and high-value secondary metabolites such as mycophenolic acid [33–37]. A CRISPR/Cas9 genome editing system has been described for *Penicillium chrysogenum,* a closely related species to *P. roqueforti*, using a similar approach as has been used for *Aspergillus* species by providing a CRISPR/Cas9 plasmid during PEG-mediated transformation [38]. This has led to the possibility of large scale genome re-engineering making *P. chrysogenum* a useful platform organism as cell factory for production of natural products [39]. Taken together, a functional CRISPR/Cas9 targeted genome editing protocol based on PEG-mediated transformations of CRISPR/Cas9 plasmids would be beneficial for future research on food spoilage capabilities and potential biotechnological advances in both *P. variotii* and *P. roqueforti.*

Many food spoiling fungi, such as *P. variotii* and *P. roqueforti*, produce asexual derived spores (conidia) that can withstand commonly used preservation treatments such as UV radiation or heat [40, 41]. Recently, the conidia of *P. variotii* have been reported to survive 60 °C for 20 min, being the most heat resistant of this type of asexual spores [10]. Additionally, conidia of food spoilage fungi are able to survive UV radiation levels used for decontamination by food industry [42–45]. It is yet unclear if pigmentation provides stress resistance against these preservation techniques in food spoiling fungi. In many ascomycetes, disruption of a specific polyketide synthase (PKS) gene results in loss of conidial pigmentation. As a consequence, these transformants produce lighter or white conidia [46–49]. Comparing the conidia of these mutants with their parental strain will lead to new insights into the potential roles of melanin in preservation stress resistance of conidia.

In this research, a functional CRISPR/Cas9 genome editing system for *P. variotii* and *P. roqueforti* is implemented to create melanin deficient mutants of both fungi, and subsequently comparing these mutants to their wild-type parental strains, using a recently described CRISPR/Cas9 deletion system developed for *A. niger* [50] with minor adaptations. This CRISPR/Cas9 genome editing system developed for *A. niger* is based on the expression of Cas9 driven from the *tef1* promoter [51]. The Cas9 expression cassette, together with the guide RNA expression cassette and the hygromycin selection marker are located on a plasmid that also contains the AMA1 sequence which enables autonomous replication in *Aspergillus* species, thereby making integration of the vector into the genome less likely [52]. This AMA1-based CRISPR/Cas9 genome editing system allows for the temporal presence of the CRISPR/Cas9 plasmid and therefore marker-free genome editing [50]. The CRISPR/Cas9 genome editing method is considerably faster than the already established marker-free genome editing method which relies on recyclable markers. The CRISPR/Cas9 based genome editing method allows for the creation of multiple mutations in a single transformation experiment, as demonstrated in *A. niger* [50], whereas the recycling method requires deletions to be performed one at a time.

Understanding the resistance mechanisms of conidia from food spoiling fungi will help us in designing novel targeted preservation techniques able to inactivate conidia without altering food flavor profiles. In order to investigate this, a working CRISPR/Cas9 genome editing system has been developed for *P. variotii* and *P. roqueforti*. These genome editing systems could enhance future research and provide a stepping stone towards creating novel biotechnologically relevant cell factories.

## Results

### Construction of melanin deficient mutants in *P. variotii* and *P. roqueforti* using CRISPR/Cas9 based genome editing.

In order to investigate the impact of melanin on stress resistance in *P. roqueforti* and *P. variotii*, melanin deficient strains in these species were made using a recently described CRISPR/Cas9 deletion system [50]. Polyketide synthase (PKS) homologues from *P. roqueforti* and *P. variotii* were identified as best bi-directional BLASTp hits with both the FwnA protein (gene ID: An09g05730) from *A. niger* [47] and the Pks17 protein (gene ID: Pc21g16000) from *P. chrysogenum* DS68530 [38]. The BLASTp searches identified Pro_LCP9604111_2|g6432.t1 as the bi-directional best hit in *P. roqueforti*. The *P. variotii* protein ID456077, recently described as PvpP in [49], was identified as the bi-directional best hit in *P. variotii*. The *P. roqueforti* protein Pro_LCP9604111_2|g6432.t1 is 68% identical to FwnA from *A. niger* and 92% identical to Pks17 from *P. chrysogenum*. The *P. variotii* protein ID456077 (PvpP) is 67% identical to FwnA and 65% identical to Pks17.

**Fig. 1:**
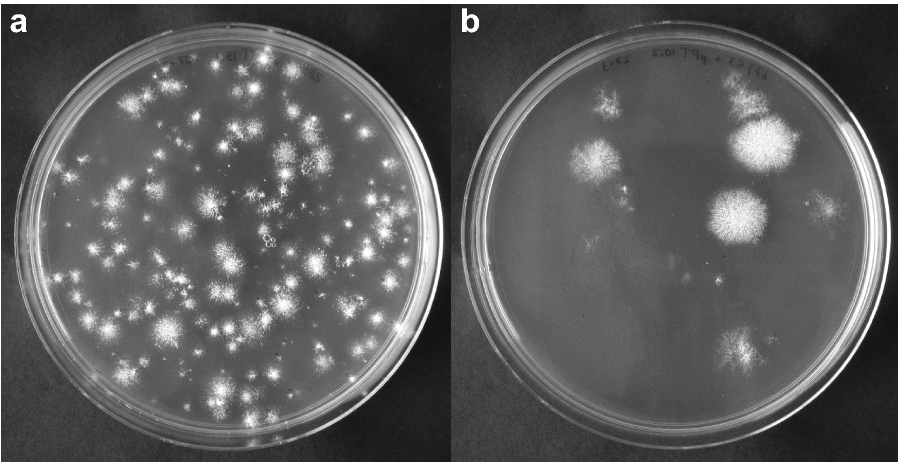
Transformation plates obtained when transforming *P. variotii* and *P. roqueforti* with PKS gene targeting CRISPR/Cas9 plasmids. **a** Transformation plate of *P. variotii* CBS101075 transformed with AMA1-based CRISPR/Cas9 plasmid pPT13.1 targeting gene *pvpP* needed for melanin biosynthesis. Typically around 100 transformants are obtained when adding 2 µg of plasmid depending on the amount of protoplasts obtained. **b** Transformation plate of *P. roqueforti* LCP9604111 transformed with AMA1-based CRISPR/Cas9 plasmid pPT9.3 targeting gene *pksA*. Typically around 10 transformants are obtained when adding 2 µg of plasmid depending on the amount of protoplasts obtained

Optimal guide RNAs for CRISPR/Cas9 genome editing for these genes were chosen based on CHOPCHOP predictors [53] (Additional file 1: Table S1). The guide RNAs were chosen to target an exon near the start codon of the open reading frame (ORF). The guide RNAs targeting the PKS homologous were cloned into the autonomously replicating vector pFC332, expressing the Cas9 nuclease, creating the CRISPR/Cas9 plasmids pPT13.1 and pPT9.3 to use for transformation of *P. variotii* and *P. roqueforti* respectively (Additional file 1: Table S1). The CRISPR/Cas9 plasmids were subsequently transformed to protoplasts of *P. variotii* CBS101075 and *P. roqueforti* LCP9604111. Transformants were obtained that produced white conidia, indicative of a disrupted melanin biosynthesis and subsequent loss of pigmentation, on the primary transformation plates for both fungi (Fig. 1). The efficiency of obtaining transformants with white conidia on the primary transformation plates in *P. variotii* and *P. roqueforti* was 83% (728 colonies with white conidia from a total of 876 transformants) and 97% (56 colonies with white conidia from a total of 58 transformants) respectively (Table 1).

**Table 1:** Gene editing efficiencies of *P. variotii* and *P. roqueforti* using CRISPR/Cas9

	*P. variotii*		*P. roqueforti*	
	*Brown conidia*	*White conidia*	*Green conidia*	*White conidia*
Phenotype obtained on first transformation plate	148/876 (17%)	728/876 (83%)	2/58 (3%)	56/58 (97%)
Hygromycin resistance loss after one round of non-selective growth	32/40 (80%)	9/40 (23%)	1/2 (50%)	16/56 (29%)

Numbers represent transformants and were calculated over multiple transformation experiments. The average amount of transformants obtained per transformation using 2 µg of CRISPR/Cas9 plasmid was ± 100 colonies for *P. variotii* and ± 10 colonies for *P. roqueforti* depending on amounts of obtained protoplasts

**Fig. 2:**
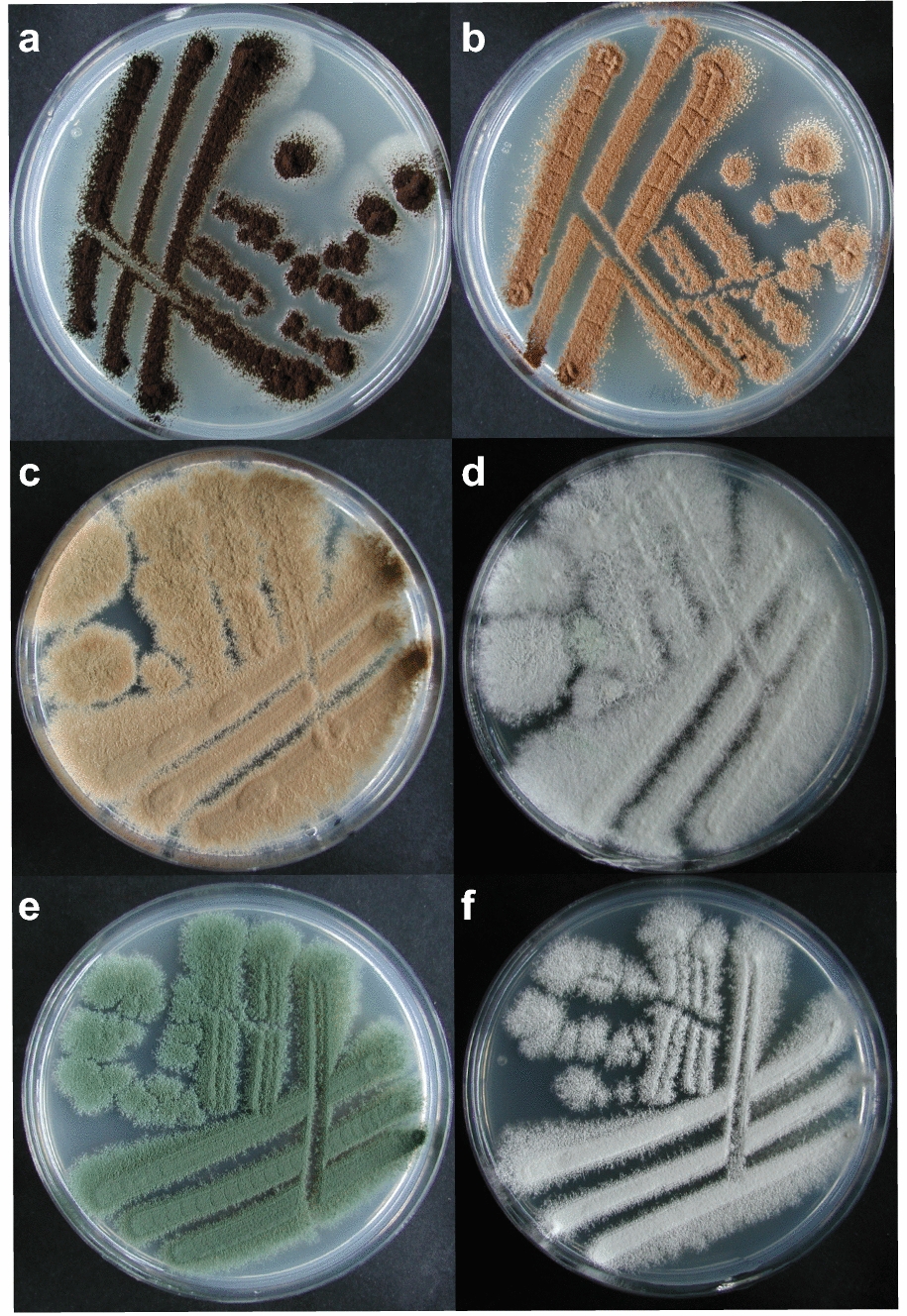
Phenotypes of parental strains and PKS mutants. **a** Phenotype *A. niger* N402, it has black conidia. **b** Phenotype *A. niger* MA93.1, it has fawn coloured / light brown conidia. **c** Phenotype *P. variotii* CBS101075 strain, it has fawn coloured conidia. **d** Phenotype *P. variotii pksA*^*−*^ strain PT32.5, it has white conidia. **e** Phenotype *P. roqueforti* LCP9604111, it has green conidia. **f** Phenotype *P. roqueforti pksA*^*−*^ strain PT34.2, it has white conidia

The efficiency of a white-coloured mutant losing hygromycin resistance in *P. variotii* and *P. roqueforti* was 23% (9 out of 40) and 29% (15 out of 56), respectively. White sporulating mutants that lost their hygromycin resistance under non-selective growth conditions were purified further and subsequently checked for mutations in the PKS genes by performing diagnostic PCRs and DNA sequencing. Melanin deficient mutants *P. roqueforti* (PT34.2) and *P. variotii* (PT32.5) were chosen for further analysis (Fig. 2). Both strains contain a 14 bps deletion in the *pksA* and *pvpP* gene respectively causing a frameshift and thus a probable genetic loss of function (Additional file 2: Figure S1). These results show that CRISPR/Cas9 genome editing using the AMA1-based expression vectors are effectively disrupting target genes in both species.

### The low efficiency of hygromycin loss after one round of non-selective growth in *P. variotii* transformants

The low efficiency of hygromycin loss in white-coloured transformants of 23% (9 out of 40) is lower compared to the efficiency of hygromycin loss in brown transformants of 80% (32 out of 40) in *P. variotii*. To investigate the low efficiency of hygromycin loss and its link with the phenotype, eleven white-coloured (of which two showed hygromcin loss) and six brown-coloured (of which four showed hygromycin loss) transformants of *P. variotii* CBS101075 were analysed by diagnostic PCR and sequencing. When analyzing the *pvpP* locus of the eleven white-coloured *P. variotii* transformants, we discovered that only 3 out of 11 (27%) mutants had the expected small indel mutation of which 1 lost the hygromycin resistant phenotype after one round of non-selective growth (Table 2). Also, 4 out of the 11 (36%) transformants analysed had part of the pPT13.1 plasmid integrated in the *pvpP* locus at the site where the double stranded break (DSB) took place, of which one lost the hygromcin resistant phenotype. The sequence and size of parts of the integrated pPT13.1 plasmid were variable. We were unable to obtain PCR products for the remaining 4 out of 11 (36%) transformants, indicating a large insertion or deletion that hampered the PCR.

**Table 2:** Plasmid integration in *P. variotii* CBS101075 transformants that did not lose hygromycin resistance

	Transformants with small indel	Transformants containing part of pPT13.1 plasmid at DBS site	No proper PCR product obtained
White conidia	3/11 (27%)	4/11 (36%)	4/11 (36%)
Brown conidia	6/6 (100%)	0/6 (0%)	0/6 (0%)

The six brown *P. variotii* transformants analysed, of which four showed hygromycin resistance loss after one round of non-selective growth, all contained an in-frame deletion in the *pvpP* locus at the DSB site (3 bps, 6 bps, 6 bps, 9 bps, 12 bps and 15 bps respectively). This indicates that the brown *P. variotii* transformants were modified by CRISPR/Cas9 gene editing, but had a functional PvpP enzyme despite the DSB and subsequent indel caused by CRISPR/Cas9.

### Construction of NHEJ repair disrupted mutants in *P. variotii* and *P. roqueforti* using CRISPR/Cas9 genome editing

In order to facilitate future genome editing in *P. variotii* and *P. roqueforti,* a *kusA*^*−*^ strain was made for both *P. variotii* and *P. roqueforti.* When performing transformations of a *kusA*^*−*^ mutant parental strain, the double stranded break caused by CRISPR/Cas9 cannot be repaired by non-homologous end-joining creating indels and instead relies on a homologous repair DNA fragment, which could be donor DNA provided by the user. This will enable targeted and complete gene knock-out or gene replacement studies. Gene deletion mutants in the *kusA* gene of *P. variotii* DTO217-A2 and *P. roqueforti* DTO013-F5 backgrounds were made, as these strains produce heat resistant conidia [10], a phenotype of interest for future food spoilage studies. The *kusA* homologous of *P. variotii* and *P. roqueforti* were identified by BLASTp analysis (best bi-directional hits) using the KusA protein from *A. niger* (An15g02700) as a query. Only a single homologous protein with significant identity score was found in *P. variotii* DTO217-A2: Pva_DTO217A2_1|g5897.t1 (66% identity). Similarly, a single homologous protein was found in *P. roqueforti*: Pro_LCP9604111_2|g3395.t1 (67% identity). Plasmids pPT23.1 and pPT22.4, containing specific guide RNA and the Cas9 expression cassettes, were made for creating *kusA*^*−*^ strains in *P. variotii* and *P. roqueforti* respectively (Additional file 1: Table S1). The guide RNAs were chosen to target an exon near the start codon of the ORFs. The creation of the *kusA*^***−***^ mutants of *P. variotii* and *P. roqueforti* relied on the creation of indels caused by NHEJ repair to disrupt the *kusA* homologous genes of these species. From 26 *P. variotii* transformants obtained only a single transformant lost the hygromycin resistant phenotype after one round of non-selective growth. In the case of *P. roqueforti* only 1 transformant was obtained and this single transformant lost the hygromycin resistance phenotype after one round of non-selective growth. Sequencing the *kusA* locus of transformants of strains *P. variotii* PT39.26 and *P. roqueforti* PT43.1 showed small indels in the *kusA* locus (7 bp deletion in the *kusA* gene of *P. variotii* and 22 bp deletion in *P. roqueforti*) resulting in frameshifts and thus potentially a disrupted *kusA* gene (Additional file 3: Figure S2).

### The impact of a disrupted NHEJ repair mechanism on the genome editing efficiency in *P. variotii* (*kusA*^*−*^)

The *P. variotii* PT39.26 (*kusA*^*−*^) strain was tested for its genome editing efficiency by transformation with the previously used pPT13.1 plasmid, which contains the *pvpP* specific guide RNA to create a double stranded break in the *pvpP* gene. A transformation of the PT39.26 strain with the pPT13.1 plasmid without providing a homologous repair DNA fragment did not give any transformants on the primary transformation plate, as expected. This indicates that indeed the NHEJ repair mechanism has been impaired in this strain and thus the strain cannot repair its double stranded break without the help of a homologous piece of DNA. Next, transformations were performed with the addition of donor DNA to allow the repair of the DSB created by the guide via homologous recombination. When donor DNA was added, putative transformants were obtained on the transformation plates. The donor DNA was a fused PCR product of both 5′ and 3′ untranslated flanks of the *pvpP* gene, which would theoretically result in the removal of the entire translated region of *pvpP* (6677 bps). In this transformation a total of fifteen transformants were obtained which all had the white-coloured phenotype. The transformants were purified from the first transformation plate and 14 out of the 15 (93%) purified transformants lost their hygromycin resistant phenotype after one round of non-selective conditions. These efficiencies are similar to those observed in *A. niger* and a major improvement over the original ratio of 1 out of 26 (3.8%) for creating a *kusA*^*−*^ strain observed in *P. variotii* DTO217-A2 or the 9 out of 40 (23%) ratio observed in *P. variotii* CBS101075. Genomic DNA was isolated for eight of these transformants. Diagnostic PCR revealed that all eight transformants were repaired using the homologous piece of DNA provided, making a full knock-out of the 6677 bps gene *pvpP* (Additional file 4: Figure S3). The PCR products of two transformants were excised from gel and subsequently send for sequencing. This confirmed repair using the HDR mechanism, replacing the original *pvpP* gene with the provided donor DNA fragment that only contained the fused flanks. In this way, we obtained the *pvpP* knock-out strain *P. variotii* PT42.1 (*kusA*^*−*^, *ΔpvpP*). All *kusA*^*−*^ mutant strains described in this study have no visible alteration in morphology and no visible change in colony diameter or radial growth rate when compared to their parental strains (Additional file 5: Figure S4).

### Heat resistance of conidia from food spoiling fungi not affected in melanin deficient mutants

**Fig. 3:**
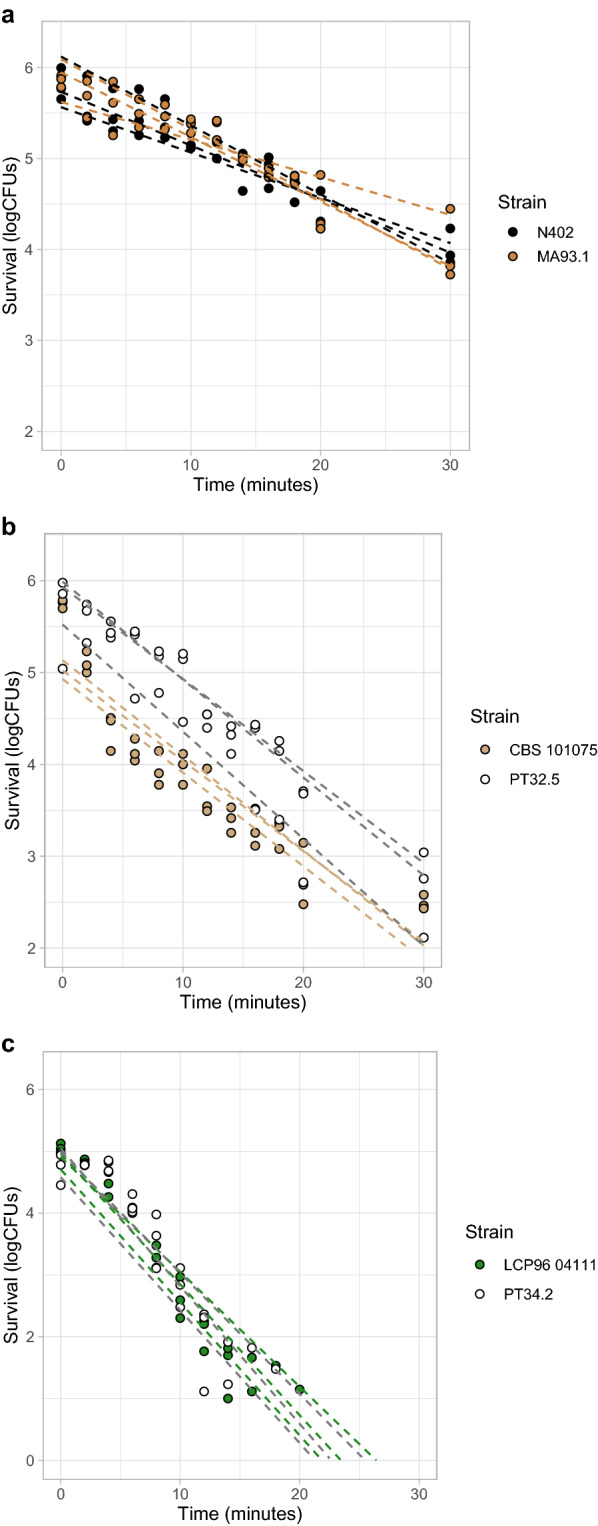
Heat resistance of three food spoiling fungi and their melanin deficient mutants. Colours used correspond with the phenotype of the conidia, see Fig. 2**a** The heat inactivation curves of *A. niger* N402 wild-type conidia (black lines) and *A. niger* MA93.1 melanin deficient mutant conidia (brown lines). The *A. niger* strains were heat treated in a 56 °C water bath. Heat inactivation shows only a 2-log reduction in microbial load for wild-type and mutant when treated for 30 min. **b** The heat inactivation curves of *P. variotii* CBS101075 wild-type conidia (light brown lines) and *P. variotii* PT32.5 melanin deficient mutant conidia (grey lines). The *P. variotii* strains were heat treated in a 60 °C water bath. Heat inactivation shows a 3-log reduction in microbial load for wild-type and mutant when treated for 30 min. **c** The heat inactivation curves of *P. roqueforti* LCP9604111 wild-type conidia (green lines) and *P. roqueforti* PT34.2 melanin deficient mutant conidia (grey lines). The *P. roqueforti* strains were heat treated in a 56 °C water bath. Heat inactivation shows at least a 5-log reduction in microbial load for wild-type and mutant when treated for 30 min. Three biological triplicates were measured. Inactivation curves were drawn based on the linear regression model

Heat inactivation assays were performed to determine the heat resistance of conidia from the *P. variotii* PT32.5 (*pvpP*^*−*^) and *P. roqueforti* PT34.2 (*pksA*^*−*^) deletion strains when compared to their parental strains. Additionally, we included the previously made *∆fwnA* strain from *A. niger* (MA93.1) and its parental strain (N402) [47]. Note that the strains with intact *kusA* genes have been used for phenotyping, as the NHEJ disruption in the *kusA*^*−*^ strains could potentially impact resistance against DNA damage caused by either UV radiation or heat. In order to observe at least a two log reduction in microbial load within 30 min, heating temperatures had to be adjusted per species. Conidia from *P. variotii* are more heat resistant than their *A. niger* and *P. roqueforti* counterparts [10, 54] and thus heat inactivation was done in a 60 °C water bath for *P. variotii* conidia instead of a 56 °C water bath for *P. roqueforti* and *A. niger*. Heat inactivation curves of wild-type and melanin deficient conidia from *A. niger, P. variotii* and *P. roqueforti* are shown in Fig. 3A, 3B and 3C respectively. Decimal reduction values were calculated based on these graphs and given in Table 3.

**Table 3:** D-values of heat inactivated conidia from three food spoiling fungi and their melanin mutants

Species and strain names		D-value ± standard deviation in time (minutes)
*A. niger*	N402	16.8 ± 3.4
*A. niger*	MA93.1	17.1 ± 6.2
*P. variotii*	CBS101075	9.3 ± 0.7
*P. variotii*	PT32.5	9.9 ± 0.3
*P. roqueforti*	LCP9604111	4.9 ± 0.4
*P. roqueforti*	PT34.2	4.7 ± 0.3

D-values are measured at 60 °C for *P. variotii* and 56 °C for *A. niger* and *P. roqueforti*

There were no significant differences in D-values based on Student’s t-tests between wild-type and mutant (all p-values were p > 0.05). Therefore, no significant difference in heat resistance between wild-type conidia and their melanin deficient mutant conidia was observed.

### UV-C radiation resistance of conidia from food spoiling fungi is affected in melanin deficient mutants

**Fig. 4:**
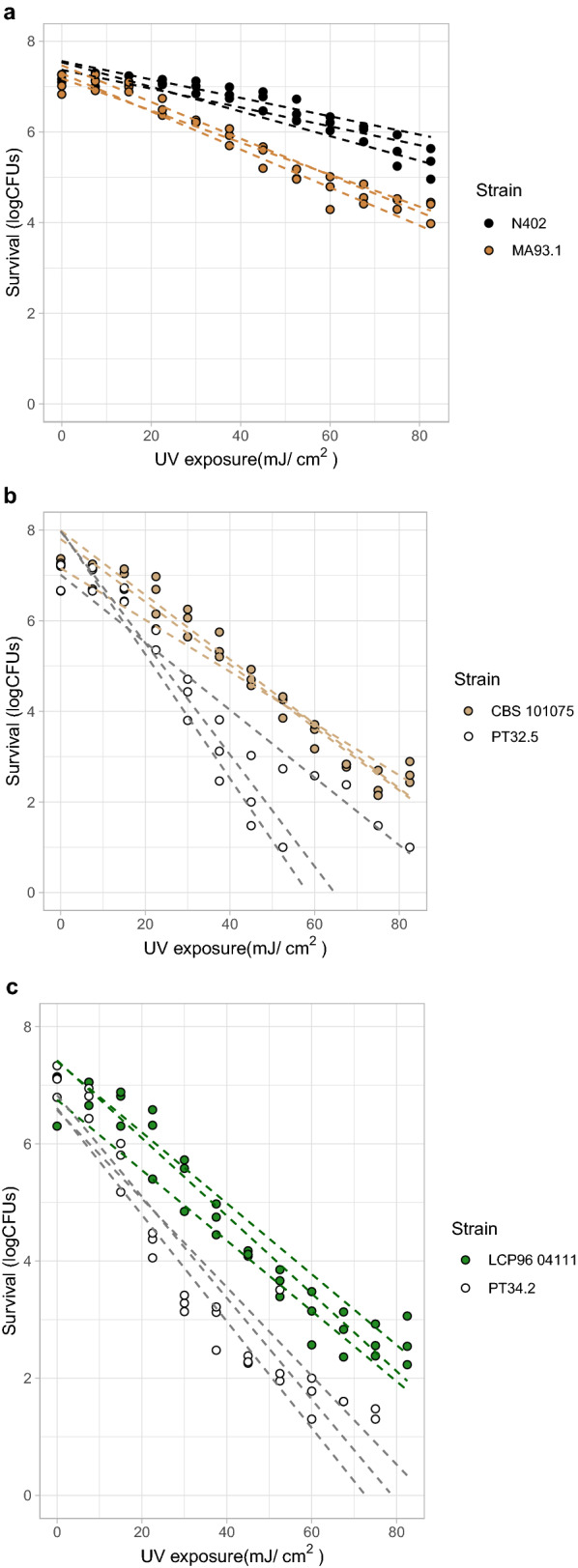
UV-C radiation resistance of melanin deficient mutants from food spoilage fungi. Colours used correspond with the phenotype of the conidia, see Fig. 2. **a** The UV inactivation curves of *A. niger* N402 wild-type conidia (black lines) and *A. niger* MA93.1 melanin deficient mutant conidia (brown lines). A maximum of 3-log reduction in microbial load was observed in the mutant after UV treatment. **b** The UV inactivation curves of *P. variotii* CBS101075 wild-type conidia (light brown lines) and *P. variotii* PT32.5 melanin deficient mutant conidia (grey lines). A maximum of 7-log reduction in microbial load was observed in the mutant after UV treatment. **c** The UV inactivation curves of *P. roqueforti* LCP9604111 wild-type conidia (green lines) and *P. roqueforti* PT34.2 melanin deficient mutant conidia (grey lines). A maximum of 7-log reduction in microbial load was observed in the mutant after UV treatment. Three biological triplicates were measured. Inactivation curves were drawn based on linear regression model

A UV-C radiation assay was performed to determine the UV resistance of the melanin deficient mutants *A. niger* MA93.1 (*ΔfwnA*), *P. variotii* PT32.5 (*pvpP*^*−*^) and *P. roqueforti* PT34.2 (*pksA*^*−*^). UV inactivation curves of conidia from *A. niger*, *P. variotii* and *P. roqueforti* are shown in Fig. 4A, 4B and 4C respectively. The results show that conidia from all three food spoiling species have reduced UV resistance when melanin biosynthesis is disrupted. Decimal reduction values were calculated to quantify this difference and are listed in Table 4.

**Table 4:** D-values of UV inactivated conidia from three food spoiling fungi and their melanin mutants

Species and strain names		D-value ± standard deviation in dose (mJ/cm^2^)
*A. niger*	N402	44.7 ± 5.6
*A. niger*	MA93.1	25.6 ± 1.9
*P. variotii*	CBS101075	15.3 ± 1.5
*P. variotii*	PT32.5	9.6 ± 2.7
*P. roqueforti*	LCP9604111	16.1 ± 0.7
*P. roqueforti*	PT34.2	11.9 ± 0.9

Student’s t-tests revealed significant UV radiation reductions when wild-type is compared to the melanin mutant in *A. niger* (p = 0.01), *P. roqueforti* (p = 0.01), but not for *P. variotii* (p = 0.06) although a similar trend is visible. The *A. niger* wild-type conidia are more resistant to UV radiation than the wild-type conidia from both *P. variotii* and *P. roqueforti* (p < 0.00 and p < 0.00 respectively). Interestingly, even the fawn coloured *A. niger* melanin mutant MA93.1 is significantly more resistant to UV than the *P. variotii* and *P. roqueforti* wild-type conidia (p < 0.00 and p < 0.00 respectively). This suggests that other melanin types or pigments in the MA93.1 strain, produced independently from the FwnA enzyme, contribute significantly to the UV radiation resistance of *A. niger* conidia. Overall, the *pks*^*−*^ mutants were more sensitive to UV radiation than their parental strains, indicating that melanin contribute to UV resistance of conidia from food spoiling fungi.

## Discussion

### CRISPR/Cas9 genome editing protocol for *P. variotii* and *P. roqueforti*

In this article a previously described CRISPR/Cas9 genome editing protocol for *A. niger* [50] was successfully implemented to perform genome editing in *P. roqueforti* and *P. variotii*. The efficiency of obtaining white-coloured transformants was 728 out of 876 (83%) in *P. variotii* CBS101075 and 56 out of 58 (97%) in *P. roqueforti* LCP9604111 (Table 1). This efficiency is comparable with previous findings in *A. niger* [50]*.* The total amount of transformants obtained per transformation experiment was consistently lower in *P. roqueforti* (n = 10 per plate) when compared to *P. variotii* (n = 100 per plate), which is probably due to less efficient protoplasting of *P. roqueforti* using adjusted *A. niger* protocols. As such, the protoplasting protocol for *P. roqueforti* should be further optimized.

### The low efficiency of hygromycin loss after one round of non-selective growth in *P. variotii* transformants

When analyzing 40 *P. variotii* CBS101075 *pvpP*^*−*^ transformants with white conidia, only 9 out of 40 (23%) lost the hygromycin resistance phenotype after one round of non-selective growth. This is in contrast to 40 *P. variotii* transformants with the wild-type brown conidia, where 32 out of 40 (80%) lost the hygromycin resistance phenotype after one round of non-selective growth. Also, when transforming *P. variotii* CBS101075 with empty vector pFC332 a similar 17 out of 24 (71%) ratio of transformants that lose their hygromycin resistant phenotype after one round of non-selective growth is obtained. In *A. niger* the reported hygromycin loss efficiency is also around 80% [50]. This suggests that having a white-coloured phenotype in *P. variotii* transformants is tied to retaining the hygromycin resistant phenotype, which could be explained by integration of the hygromycin resistance marker on the *pvpP* locus itself where the double stranded break occurs. Indeed, further investigation of *P. variotii* transformants revealed that a significant portion of at least 4 out of 11 (36%) transformants with white conidia contained pieces of the pPT13.1 plasmid at the site where the double stranded break had occurred (Table 2). This observation suggests that the double stranded break in the genomic DNA of *P. variotii* is somehow repaired using the CRISPR/Cas9 containing vector itself. The integration of the AMA1-based vector containing the hygromycin selection marker explains the stability of the hygromycin marker even under non-selective conditions in *P. variotii*. Since the hygromycin loss efficiency in *P. roqueforti* is also low (29%, see Table 1), we speculate that the same plasmid integration is happening in *P.roqueforti* transformations.

Obtaining a transformant with a mutation in the targeted gene is highly efficient, thus this methodology suffices when a single gene disrupted mutant is made. However, the high stability of the hygromycin marker limits the possibility for sequential rounds of transformations. Additionally, this methodology does not allow for efficiently obtaining full knock-out mutants, nor the efficient targeted integration of new pieces of DNA (for example to produce heterologous proteins) as the NHEJ repair mechanism is the preferred DNA repair mechanism over HDR in most filamentous fungi [6]. Disrupting the NHEJ repair mechanism is therefore beneficial as it could potentially circumvents all these drawbacks. Therefore, the created *kusA*^*−*^ mutants that are incapable of performing NHEJ are valuable tools for future genome editing in both *P. variotii* and *P. roqueforti*.

### The impact of a disrupted NHEJ repair mechanism on the genome editing efficiency in *P. variotii* (*kusA*^*−*^)

It was noticed that in the process of obtaining the *kusA*^*−*^ mutant in *P. variotii* DTO217-A2, only 1 out of 26 (3%) of transformants lost the hygromycin resistance phenotype after one round of non-selective growth. This again stresses the inefficiency of obtaining a marker free mutant after one round of growth on non-selective media when transforming a *kusA*^+^ wild-type *P. variotii* strain with a AMA1-based CRISPR/Cas9 plasmid as previously seen when isolating melanin mutants (Table 1). After obtaining this single *P. variotii* PT39.26 (*kusA*^*−*^) strain, we investigated its genome editing efficiency. *P. variotii* PT39.26 (*kusA*^*−*^) was transformed using the pPT13.1 plasmid (pFC332 containing a *pvpP* targeting sgRNA) together with the addition of donor DNA consisting of the fused flanks of the *pvpP* gene. Obtaining white-coloured transformants in this transformation was highly efficient as 15 out of the 15 transformants produced white spores. The percentage of these transformants that lost their hygromycin resistant phenotype after one round of non-selective growth was also highly efficient, 14 out of 15 (93%). This is a major improvement over the 1 out of 26 (3%) ratio of hygromycin resistance loss mentioned earlier when creating the *kusA*^*−*^ strain. Further analysis of eight of these white-coloured *P. variotii* transformants revealed that all eight transformants lost the complete *pvpP* gene (6677 bps) as checked by diagnostic PCR (Additional file 4: Figure S3), indicating that repair by HDR had taken place. This was further confirmed by sequencing 2 out of the 8 transformants which showed the knock-out of the complete 6677 bps as expected. Thus, eight knock-out mutants were obtained where no chunks of pPT13.1 plasmid were integrated into the *pvpP* locus. Taken together, the increased efficiency in hygromycin resistance loss and the absence of pPT13.1 chunks on the *pvpP* locus in transformants obtained from the *P. variotii* PT39.26 *kusA*^*−*^ strain indicate that by disrupting the NHEJ repair mechanism in *P. variotii* DTO217-A2 the integration of the AMA1 containing vector into the genome is prevented or severely reduced. Therefore, we conclude that the high degree of plasmid integration into the target site where DSB took place was due to repair facilitated by the NHEJ mechanism. Increasing overall efficiency of obtaining genetic alterations by HDR will open up future research for generating full knock-out mutants as well as targeted integration of DNA (e.g. fluorescent proteins or non-native enzymes). The *kusA*^*−*^ strains can be safely used for strain development as genome stability is not severely altered in NHEJ disrupted filamentous fungi [55].

In future research, it could be preferred to restore the *kusA* locus after complete knock-out of the target genes has been confirmed. For example, conidia of the *ΔkusA* mutant of *A. niger* show increased sensitivity to UV radiation resistance [8]. Thus, in order to investigate the impact of *pvpP* in the UV-C radiation resistance of conidia obtained from *P. variotii* PT42.1 (*kusA*^*−*^, *ΔpvpP*) it would be desirable to restore the *kusA* gene back to wild-type. This can be done by performing the same CRISPR/Cas9 genome editing system described for the creation of the *ΔpvpP*, but changing the target sequence to target the disrupted *kusA* locus and provide donor DNA with the intact wild-type *kusA* gene and its flanks. However, the restored wild-type *kusA* gene should not be recognized by the guide RNA targeting the disrupted *kusA* locus. This can be achieved by either targeting the indel itself, or incorporating silent point mutations in the donor DNA.

### The contribution of melanin on the heat resistance and UV radiation resistance of conidia in three food spoiling fungi

There have been reports on yeast species in which melanization correlates to heat resistance [56, 57]. However, no altered heat resistance was observed in the conidia of the three food spoiling filamentous fungi with disrupted melanin biosynthesis. It has been previously shown that the conidia of *A. niger* strain MA93.1 have no altered heat inactivation resistance when compared to the parental strain N402 [58]. Here we similarly show no heat inactivation alterations for the mutant strains *P. roqueforti* PT34.2 and *P. variotii* PT32.5 when compared to their parental strains. These results suggests that melanin does not play a significant role in conidial heat resistance in these food spoiling fungi. In contrast, conidia disrupted in their melanin production showed increased susceptibility to UV-C radiation in all three food spoiling fungi. The UV-C radiation resistance of *A. niger* MA93.1 is lowered compared to the parental strain, as was previously shown [58]. Additionally, here we show that a gene disruption in the *pksA* homologue of *P. roqueforti* LCP9604111 and the *pvpP* gene in *P. variotii* CBS101075 also reduce UV-C radiation resistance of the conidia from these mutants. Interestingly, this result is in contrast to the polyketide synthase (*alb1*) knockdown mutant of *Penicillium marneffei,* which did not show altered UV-C radiation resistance [48]. It is apparent that the melanin deficient *A. niger* MA93.1 strain is more UV-C radiation resistant than either *P. roqueforti* or *P. variotii* wild-type strains (p < 0.00 student’s t-test). This suggests that the remaining pigmentation in the *A. niger* MA93.1 strain is a significant factor in UV-C radiation resistance, explaining the significant differences in resistance between *A. niger* and both the *P. roqueforti* and *P. variotii* strains (Fig. 4). The *A. niger* conidia seem to have an additional type of pigmentation that does not require the functionality of the FwnA enzyme. Which type of pigmentation still remains in *A. niger* MA93.1 is currently unknown. There are reports of several *Aspergillus* species producing other melanin types besides DHN-melanin, such as DOPA-melanin or pyomelanin [59, 60], which would make likely candidates. A total of eight different melanin types have been described for fungi alone, each with their own distinct biosynthesis pathway [61]. The relation between the type of fungal melanin and subsequent UV radiation and heat resistance is currently unknown. Additionally, we noted that UV radiation resistance of the three melanin deficient mutants is about two-thirds of their wild-type parental strains in each species. This finding suggests that the relative contribution of the PKS produced pigmentation to the UV radiation resistance of conidia is similar in each species.

## Conclusions

We have shown the successful implementation of the AMA1-based CRISPR/Cas9 genome editing system in *P. variotii* and *P. roqueforti*, which is capable of creating indels in the targeted gene. However, we observed a large amount of plasmid integration events when using the AMA1-based plasmids in this way, resulting in mutant strains which are often no longer marker-free. We demonstrate that a *kusA*^*−*^ background can be used to prevent or otherwise severely reduce plasmid integration, which allows for efficient marker-free genome editing and additionally facilitates the creation of complete knock-outs by relying on the homology directed DNA repair mechanism.

We have used the AMA1-based CRISPR/Cas9 plasmids to create melanin deficient mutants of *P. variotii* and *P. roqueforti*, in order to analyse their preservation stress resistance*.* We show that the melanin-lacking conidia of food spoilers *P. variotii*, *P. roqueforti* and *A. niger* are not altered in their heat resistance compared to their parental strains. In contrast, mutant conidia of food spoilers *P. variotii*, *P. roqueforti* and *A. niger* have increased sensitivity towards UV-C radiation. As such, the presence of DHN-melanin in conidia of three food spoiling fungi does not contribute to their heat resistance, but does contribute to their UV-C radiation resistance.

## Materials and methods

### Strains, growth conditions, spore harvesting, media and molecular techniques

The *A. niger*, *P. roqueforti* and *P. variotii* strains used in this study are listed in Table 5.

**Table 5:** Strains used in this study

Strain name	Genotype	Reference
*Aspergillus niger* N402	*cspA1*	[62]
*Aspergillus niger* MA93.1	*cspA1, fwnA::hygB* in N402	[47]
*Paecilomyces variotii* CBS101075	wild-type	[22]
*Paecilomyces variotii* PT32.5	*pvpP*^*−*^ in CBS101075	This study
*Paecilomyces variotii* DTO217-A2	wild-type	[10]
*Paecilomyces variotii* PT39.26	*kusA*^*−*^ in DTO217-A2	This study
*Paecilomyces variotii* PT42.1	*kusA*^*−*^*, ΔpvpP* in DTO217-A2	This study
*Penicillium roqueforti* LCP9604111	wild-type	[54]
*Penicillium roqueforti* PT34.2	*pksA*^*−*^ in LCP9604111	This study
*Penicillium roqueforti* DTO013-F5	wild-type	Westerdijk Fungal Biodiversity Institute, CBS collection
*Penicillium roqueforti* PT43.1	*kusA*^*−*^ in DTO013-F5	This study

The *Escherichia coli* strain DH5α was used for cloning purposes. Fungal strains were grown for 7 days at 25 °C on malt extract agar (MEA) unless noted otherwise. All media used and spore harvesting methods are described by Arentshorst et al. [63]. Standard PCR and *E. coli* cloning techniques were used according to Sambrook et al. [64]. Spore suspensions were made using physiological salt buffer (0.9% NaCl + 0.02% Tween 80 in demiwater) unless noted otherwise. The *P. roqueforti* strains were harvested and washed in ACES buffer (10 mM N‐(2‐acetamido)‐2‐aminoethanesulfonic acid, 0.02% Tween 80, pH 6.8) according to van den Brule et al. [10].

### CRISPR/Cas9 plasmids construction

All plasmids and primers used in this study are listed in the supplementary data (Additional file 1: Table S1, Additional file 6: Table S2). In silico work was performed on FASTA files obtained from JGI [65]. Plasmid construction was based on earlier work performed in *A. niger* [50]. A detailed protocol on the CRISPR/Cas9 plasmid construction and subsequent transformations in *P. variotii* and *P. roqueforti* can be found in the supplementary data (Additional file 7: Protocol S1). Briefly, the plasmids pTLL108.1 and pTLL109.2 were used as templates for creating the 5′ flank and 3′ flank of the sgRNA respectively. After fusion PCR using the pTE1_for and pTE1_rv primers, a PacI (Fermentas, Thermo Scientific™) digestion on the purified PCR product was performed O/N. The PacI digested sgRNA was ligated into a PacI digested and dephosphorylated pFC332 plasmid and subsequently cloned into *E. coli* DH5α. The ampicillin resistant colonies were grown under selective pressure overnight and miniprepped (GeneJET Plasmid Miniprep Kit, Thermo Scientific™), after which restriction analysis was done with SacII (Fermentas, Thermo Scientific™) to check for correct insertion of the sgRNA. Lastly, sequencing was performed as a final check to ensure correct sgRNA is present in the newly constructed plasmid.

### Transformation protocol and DNA isolation

Fungal transformations were performed according to van Leeuwe et al. [50] with a few adaptations. Hygromycin concentrations used for selection during transformation were adjusted per species, chosen based on the lowest concentration still preventing background growth. As such, the final concentrations used were 100 µg/ml hygromycin for *P. roqueforti* transformations and 200 µg/ml hygromycin for *P. variotii* transformations. Since these wild-type strains were *kusA*^+^, gene disruptions relied on non-homologous end joining (NHEJ) for repair resulting in the creation of indels, see van Leeuwe et al. [50] for more information. In contrast, after the *P. variotii* PT39.26 (*kusA*^*−*^) strain was obtained transformation with pPT13.1 was done with the addition of a repair DNA fragment to obtain *P. variotii* PT42.1 (*kusA*^*−*^, *ΔpvpP*), see Results section. The mycelium for protoplasting and subsequent transformation of *P. roqueforti* was pre-grown in CM for 2 days at 25 °C instead of 1 day at 30 °C for *A. niger* and *P. variotii*. Protoplasting was done in SMC medium with Lysing enzymes (Sigma) essentially as described previously [63]. Protoplast formation was checked by light microscopy every 15 min for both *P. roqueforti* and *P. variotii*. The protoplasting process was commonly stopped after 45 min, when protoplasts were visually present. Genomic DNA isolations were done according to Arentshorst et al. [63].

### Heat resistance assay

The heat inactivation assay was based on van den Brule et al. [10] with few exceptions. At t = 0 a total of 200 µl spore suspension of 1*10^8 conidia/ml was added to pre-heated 19.8 mL ACES buffer (*P. roqueforti*) or 19.8 mL physiological salt buffer (*P. variotii* and *A. niger*). The temperatures of the water bath were adjusted per species (56 °C *A. niger*, 56 °C *P. roqueforti*, 60 °C *P. variotii*). The *P. variotii* and *A. niger* conidia were treated in a static water bath with magnetic stirring (Julabo Corio C-BT19) at 180 rpm inside 50 ml Erlenmeyers. The *P. roqueforti* conidia were treated in a shaking water bath (Grant OLS200) at 100 rpm. Samples were taken every 2 min until t = 20 min. Additionally t = 30 min was taken as a final sample. Heat inactivation curves and standard deviations were made based on three biological replicates. Dilutions were made in either ACES buffer (*P. roqueforti*) or physiological salt buffer (*P. variotii* and *A. niger*) corresponding with their heating menstruum. Spore suspensions were plated on MEA plates for colony counting. The colony forming units (CFUs) were counted after 7 days of growth at 25 °C. Decimal reduction values (*D*-values) were calculated using the linear regression model.

### UV-C radiation resistance assay

The UV-C radiation resistance assay was done in a UV crosslinker (Hoefer UVC 500 Ultraviolet Croslinker). A total of 2*10^7 conidia per mL were UV exposed inside open Petri dishes (total starting volume = 25 mL). After each UV dose, 1 mL of spore suspension was taken and subsequently serially diluted and plated on MEA plates. Survival was measured by CFUs scored after 7 days. The lowest dose applied was 7.5 mJ/cm^2^ and then increased by 7.5 mJ/cm^2^ in a stepwise manner with a maximum dose of 82.5 mJ/cm^2^. The UV radiation resistance assays were performed in biological triplicates. Decimal reduction values (D-values) were calculated based on the linear regression model. Significance was tested using an unpaired Student’s *t*-test (significant if *p* < 0.05).

## Supplementary Information

**Additional file 1: Table S1.** Plasmids used in this study.

**Additional file 2: Figure S1.** Indels found in *P. variotii* PT32.5 and *P. roqueforti* PT34.2. Sequencing results of pksA genes after mutation caused by CRISPR/Cas9 double stranded break and subsequently repair with the NHEJ repair machinery.**a** Indel of 14 bps in the pvpP gene in *P. variotii* causing a frameshift soon after the start codon. This frameshift likely disrupts gene function. **b** Indel of 14 bps in the pksA gene of *P. roqueforti* causing a frameshift soon after the start codon. This frameshift likely disrupts gene function. Pictures were made using Benchling [Biology Software] (2020). Retrieved from https://benchling.com.

**Additional file 3: Figure S2.** Indels found in *kusA*^*−*^ strains *P. variotii* PT39.26 and *P. roqueforti* PT43.1.Sequencing results of kusA genes after mutation caused by CRISPR/Cas9 double stranded break and subsequently repair with the NHEJ repair machinery. **a** Indel of 7 bps in the kusA gene in *P. variotii* PT39.26 causing a frameshift soon after the start codon. This frameshift likely disrupts gene function. **b** Indel of 22 bps in the kusA gene of *P. roqueforti* PT43.1 causing a frameshift soon after the start codon. This frameshift likely disrupts gene function. Pictures were made using Benchling [Biology Software] (2020). Retrieved from https://benchling.com.

**Additional file 4: Figure S3.** Diagnostic PCR on eight transformants in *P. variotii* PT39.26 missing the *pvpP* gene. **a** Diagnostic PCR to investigate the presence of the pvpP gene by amplifying outside the used flanks. If the gene is absent a band size of 2619 bps is expected. If the gene pvpP is still present, a band size of 9344 bps is present. The eight transformants all have lost the pvpP gene. The PCR fragments loaded on #6 and #7 were purified and subsequently send for sequencing. **b** Diagnostic PCR to investigate the presence of the pvpP gene by amplifying inside the gene. If the gene is absent, no band is expected. In wild-type situation, a PCR fragment of 1095 bps is expected. No transformants show the presence of pvpP gene. Taken together, these results show that 8/8 transformants had a full knock-out of the pvpP gene. Both contained the expected sequence for the repair DNA fragment, indicating repair by HDR.

**Additional file 5: Figure S4.** Growth rate of *P. variotii* and *P. roqueforti* strains. Colony diameters were measured of *P. variotii* and *P. roqueforti* strains growing 2-5 days at 25°C point-inoculated on MEA plates. **a** Colony diameters of strains plotted against time. The average growth rate in millimeters was estimated by performing a best fit using linear regression. The slope represents the average millimeter increase in diameter per day. On average, all *P. variotii* strains increased 18-19 mm in diameter per day and all P. roqueforti strains increased 10-11 mm in diameter per day. No differences were observed between mutant strains and their parental strains. **b** Morphology of *P. roqueforti* strains growing on MEA plates. No differences are visible between mutant strains and their parental strains except for the expected change in spore coloration. **c** Morphology of *P. variotii* strains growing on MEA plates. No differences are visible between mutant strains and their parental strains except for the expected change in spore coloration

**Additional file 6: Table S2.** Primers used in this study.

**Additional file 7. Protocol** **S1.** A detailed protocol for CRISPR/Cas9 mediated gene knock-out in *Penicillium roqueforti* and *Paecilomyces variotii*.

## Data Availability

All datasets supporting the results of this article are included within the article or in the additional files. Plasmids and strains are available upon request. Strains have been deposited at the KNAW strain archive.
