# Supplementary material for: Preservation stress resistance of melanin deficient conidia from Paecilomyces variotii and Penicillium roqueforti mutants generated via CRISPR/Cas9 genome editing
Source: Fungal Biol Biotechnol. 2021 Apr 2;8:4. doi: 10.1186/s40694-021-00111-w (PMC8017634; doi:10.1186/s40694-021-00111-w)
Supplement: Supplementary file 1 — Additional file 1: Table S1. Plasmids used in this study. [file 40694_2021_111_MOESM1_ESM.docx]

| Plasmid name | Parental plasmid | Gene number of target gene | Gene name | Target sequence | Reference |
| --- | --- | --- | --- | --- | --- |
| pFC332 | - | - | - | - | [41] |
| pPT9.3 | pFC332 | Pro_LCP9604111_2\|g6432.t1 | *pksA* | GTCTTGATGCCACAGTGAGG | This study |
| pPT13.1 | pFC332 | ID456077 | *pvpP* | GGCTTCTCGACATTGATCGG | This study |
| pPT22.4 | pFC332 | Pro_LCP9604111_2\|g3395.t1 | *kusA* | CTATATTCTTAAATGGGCGG | This study |
| pPT23.1 | pFC332 | ID464258 | *kusA* | GCAGGCTCTCGCATTAGACG | This study |

**Additional file 5. Table S1.** All plasmids used in this study
