# Supplementary figures and images for: Preservation stress resistance of melanin deficient conidia from Paecilomyces variotii and Penicillium roqueforti mutants generated via CRISPR/Cas9 genome editing
Source: Fungal Biol Biotechnol. 2021 Apr 2;8:4. doi: 10.1186/s40694-021-00111-w (PMC8017634; doi:10.1186/s40694-021-00111-w)

**A.**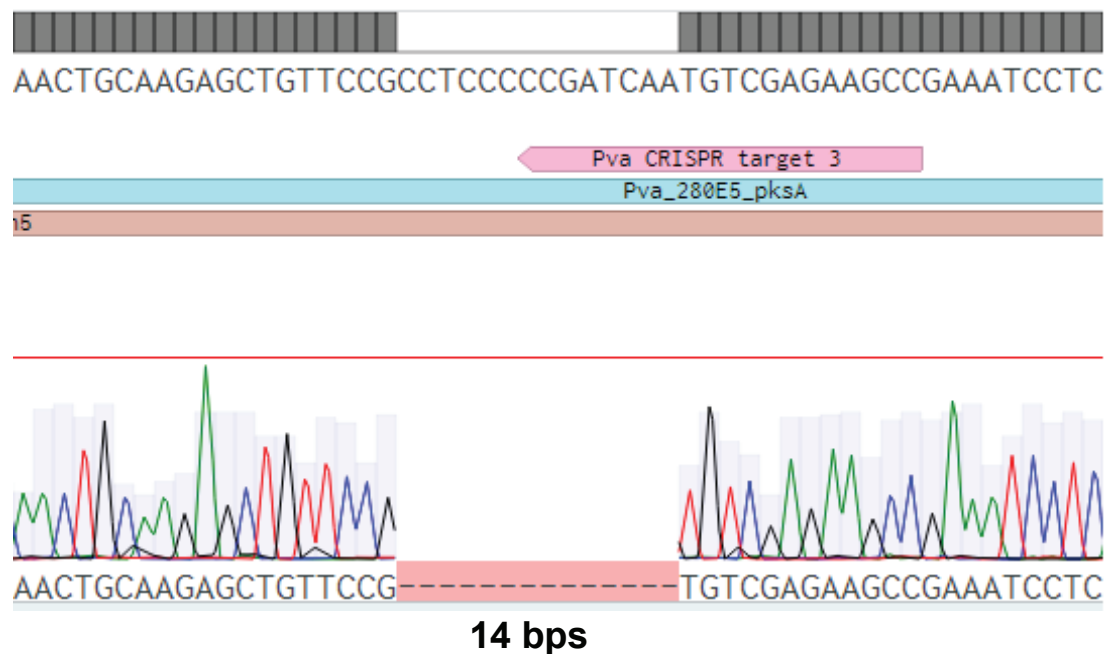**B.**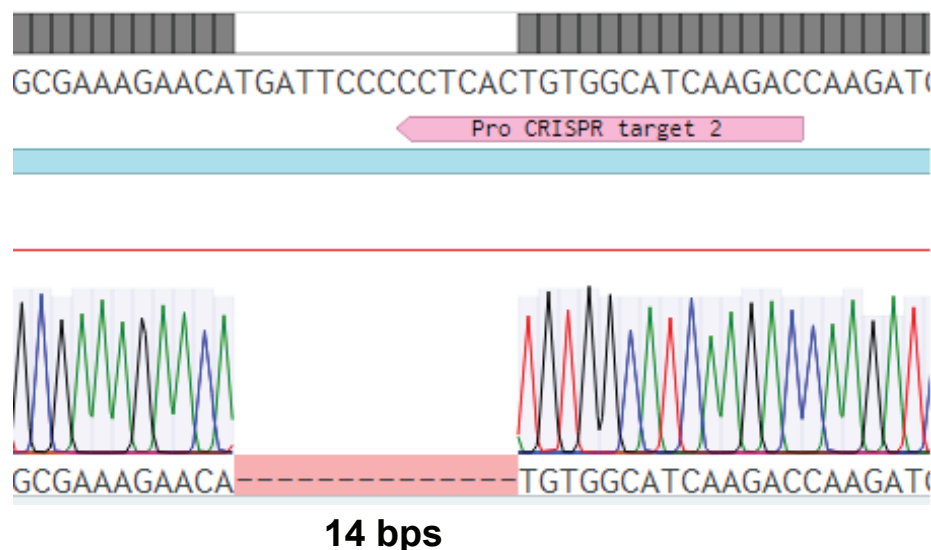

Supplement: Supplementary file 2 — Additional file 2: Figure S1. Indels found in P. variotii PT32.5 and P. roqueforti PT34.2. Sequencing results of pksA genes after mutation caused by CRISPR/Cas9 double stranded break and subsequently repair with the NHEJ repair machinery.a Indel of 14 bps in the pvpP gene in P. variotii causing a frameshift soon after the start codon. This frameshift likely disrupts gene function. b Indel of 14 bps in the pksA gene of P. roqueforti causing a frameshift soon after the start codon. This frameshift likely disrupts gene function. Pictures were made using Benchling [Biology Software] (2020). Retrieved from https://benchling.com. [file 40694_2021_111_MOESM2_ESM.pdf]

A.

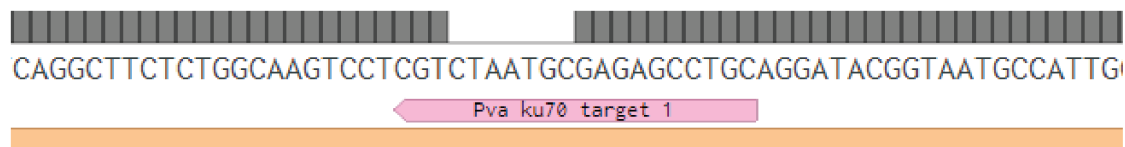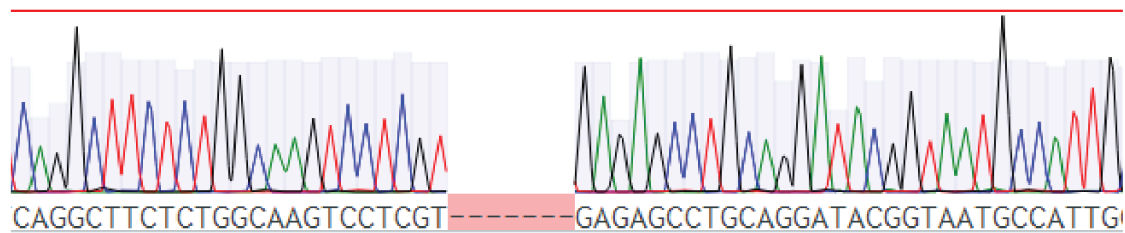

7 bps

B.

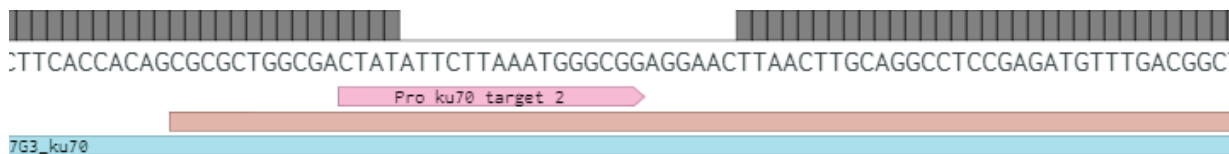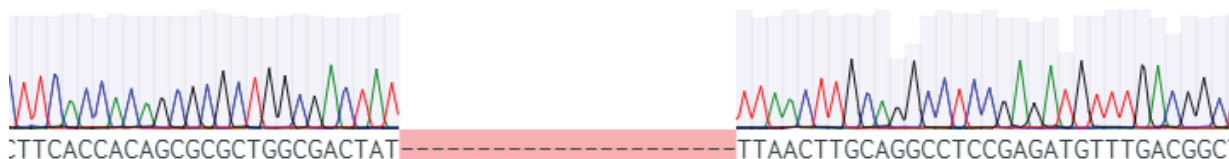

22 bps

Supplement: Supplementary file 3 — Additional file 3: Figure S2. Indels found in kusA− strains P. variotii PT39.26 and P. roqueforti PT43.1.Sequencing results of kusA genes after mutation caused by CRISPR/Cas9 double stranded break and subsequently repair with the NHEJ repair machinery. a Indel of 7 bps in the kusA gene in P. variotii PT39.26 causing a frameshift soon after the start codon. This frameshift likely disrupts gene function. b Indel of 22 bps in the kusA gene of P. roqueforti PT43.1 causing a frameshift soon after the start codon. This frameshift likely disrupts gene function. Pictures were made using Benchling [Biology Software] (2020). Retrieved from https://benchling.com. [file 40694_2021_111_MOESM3_ESM.pdf]

A.

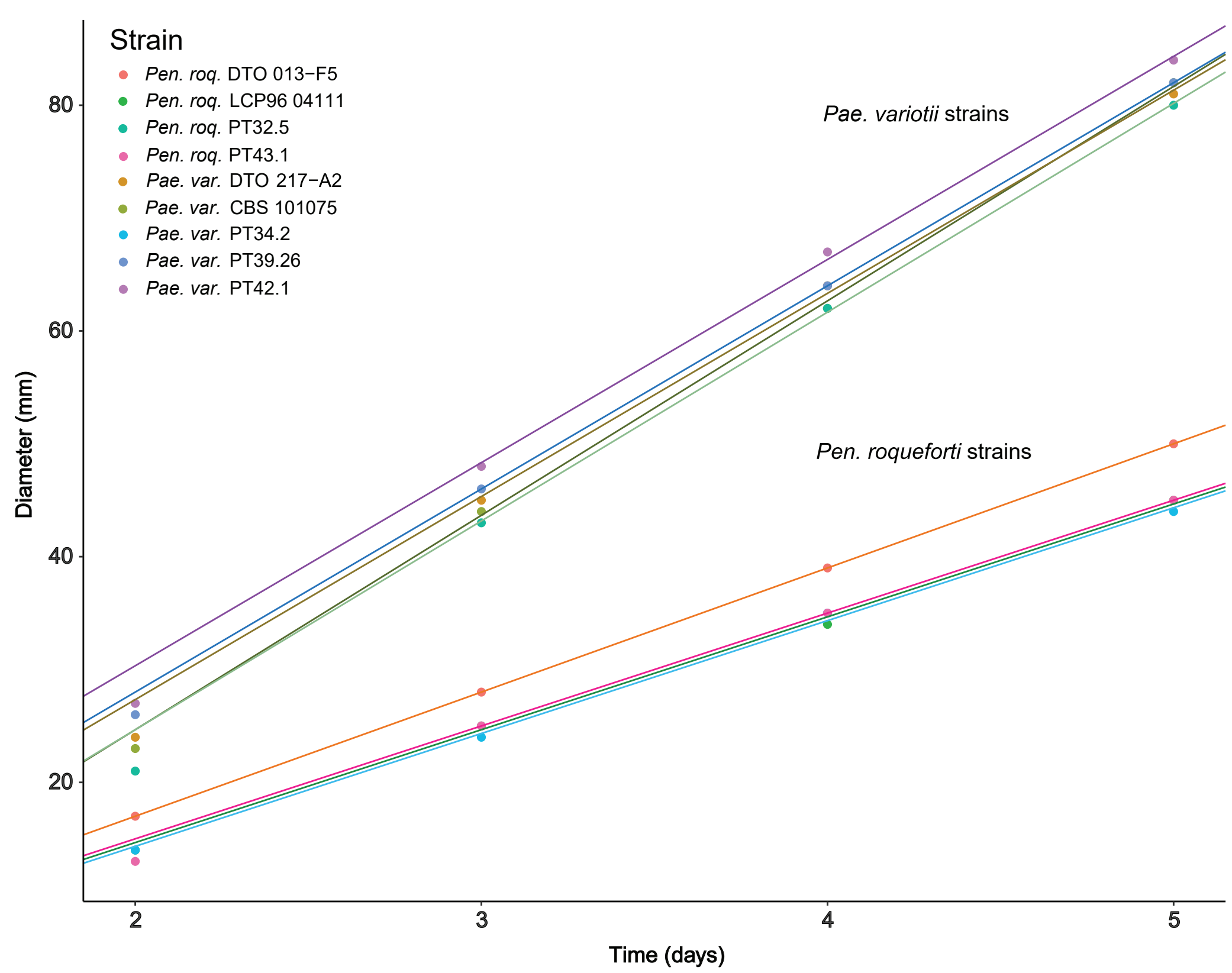

B.

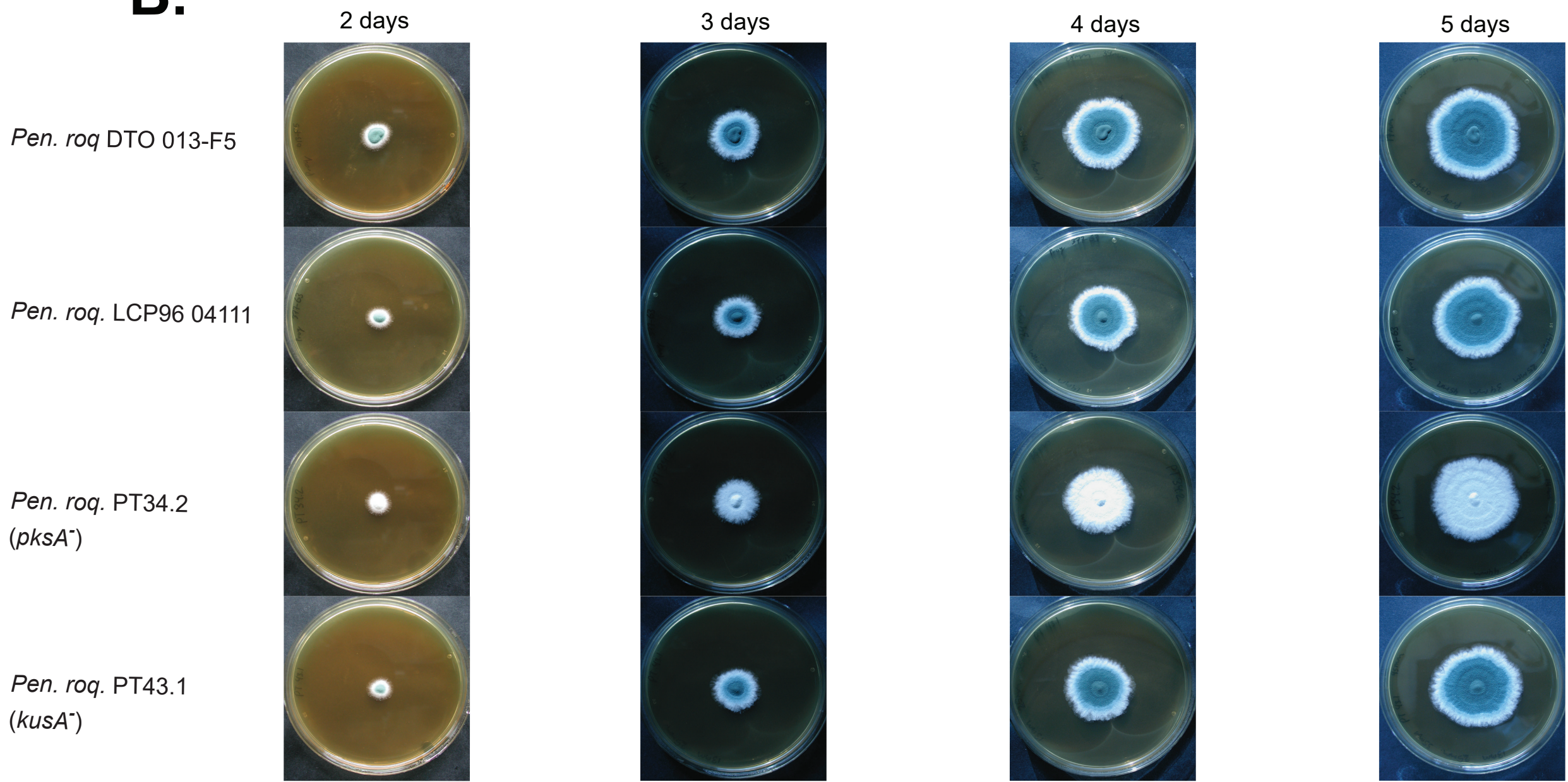

C.

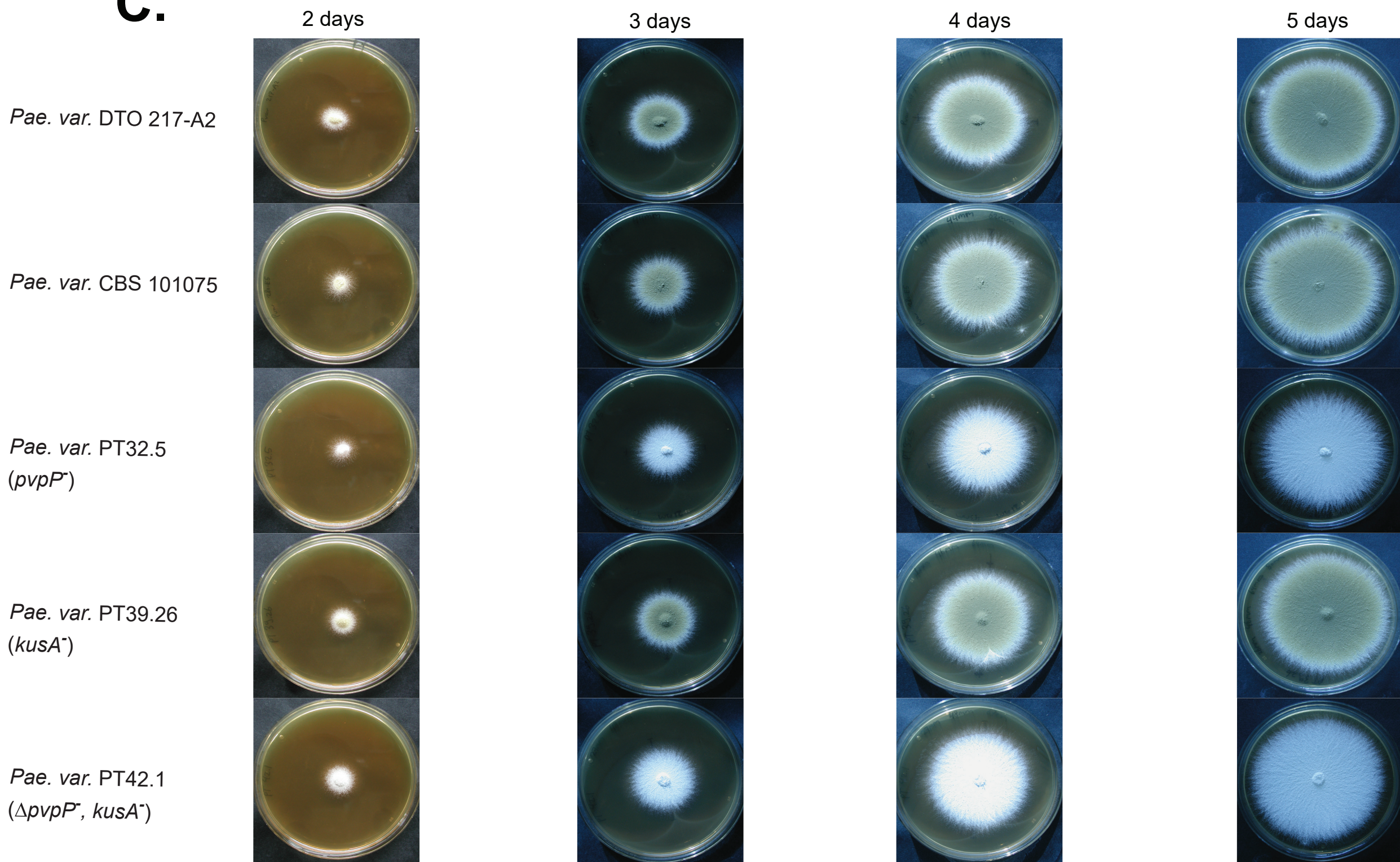

Supplement: Supplementary file 5 — Additional file 5: Figure S4. Growth rate of P. variotii and P. roqueforti strains. Colony diameters were measured of P. variotii and P. roqueforti strains growing 2-5 days at 25°C point-inoculated on MEA plates. a Colony diameters of strains plotted against time. The average growth rate in millimeters was estimated by performing a best fit using linear regression. The slope represents the average millimeter increase in diameter per day. On average, all P. variotii strains increased 18-19 mm in diameter per day and all P. roqueforti strains increased 10-11 mm in diameter per day. No differences were observed between mutant strains and their parental strains. b Morphology of P. roqueforti strains growing on MEA plates. No differences are visible between mutant strains and their parental strains except for the expected change in spore coloration. c Morphology of P. variotii strains growing on MEA plates. No differences are visible between mutant strains and their parental strains except for the expected change in spore coloration [file 40694_2021_111_MOESM5_ESM.pdf]
