# Supplementary material for: Preservation stress resistance of melanin deficient conidia from Paecilomyces variotii and Penicillium roqueforti mutants generated via CRISPR/Cas9 genome editing
Source: Fungal Biol Biotechnol. 2021 Apr 2;8:4. doi: 10.1186/s40694-021-00111-w (PMC8017634; doi:10.1186/s40694-021-00111-w)

A.

Primer combination Pva_pksAP22f and Pva_pksAP23r

Expected band sizes mutant: 2619 bps

wild-type: 9344 bps (too large so no band)

transformants #1-#8
Wild-type

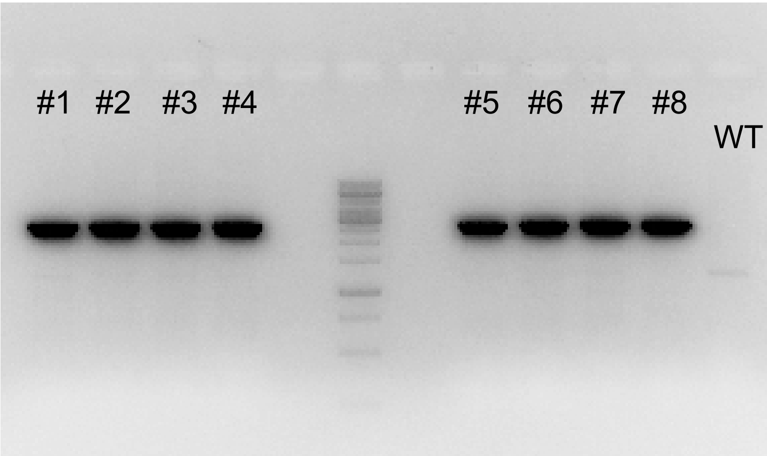

B.

Primer combination Pva_pksAP19f and Pva_pksAP20r

Expected band sizes mutant: - (no gene present)

wild-type: 1095 bps

transformants #1-#8
Wild-type

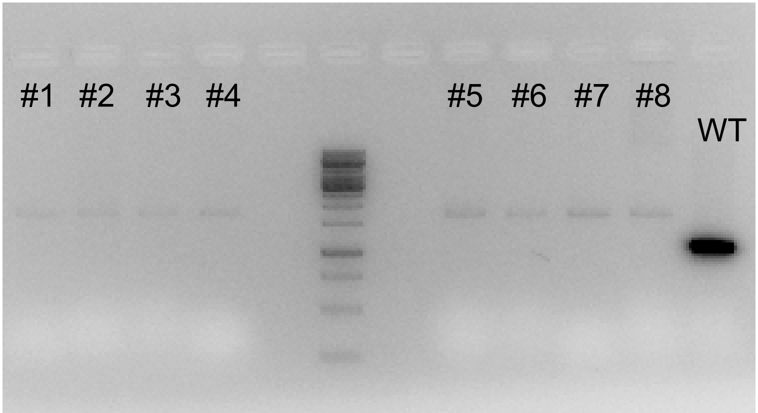

Supplement: Supplementary file 4 — Additional file 4: Figure S3. Diagnostic PCR on eight transformants in P. variotii PT39.26 missing the pvpP gene. a Diagnostic PCR to investigate the presence of the pvpP gene by amplifying outside the used flanks. If the gene is absent a band size of 2619 bps is expected. If the gene pvpP is still present, a band size of 9344 bps is present. The eight transformants all have lost the pvpP gene. The PCR fragments loaded on #6 and #7 were purified and subsequently send for sequencing. b Diagnostic PCR to investigate the presence of the pvpP gene by amplifying inside the gene. If the gene is absent, no band is expected. In wild-type situation, a PCR fragment of 1095 bps is expected. No transformants show the presence of pvpP gene. Taken together, these results show that 8/8 transformants had a full knock-out of the pvpP gene. Both contained the expected sequence for the repair DNA fragment, indicating repair by HDR. [file 40694_2021_111_MOESM4_ESM.pdf]
