# Supplementary material for: Preservation stress resistance of melanin deficient conidia from Paecilomyces variotii and Penicillium roqueforti mutants generated via CRISPR/Cas9 genome editing
Source: Fungal Biol Biotechnol. 2021 Apr 2;8:4. doi: 10.1186/s40694-021-00111-w (PMC8017634; doi:10.1186/s40694-021-00111-w)
Supplement: Supplementary file 6 — Additional file 6: Table S2. Primers used in this study. [file 40694_2021_111_MOESM6_ESM.docx]

**Additional file 6. Table S2.** All primers used in this study

| Primer name | Sequence | Used for |
| --- | --- | --- |
| pTE1_for | CCTTAATTAAACTCCGCCGAACGTACTG | Creating new CRISPR gRNAs |
| pTE1_rev | CCTTAATTAAAAAAGCAAAAAAGGAAGGTACAAAAAAGC | Creating new CRISPR gRNAs |
| Pro_pks1P9r | CCTCACTGTGGCATCAAGACGACGAGCTTACTCGTTTCG | Creating CRISPR/Cas9 target for *pksA* gene in *P. roqueforti* |
| Pro_pks1P10f | GTCTTGATGCCACAGTGAGGGTTTTAGAGCTAGAAATAGCAAG | Creating CRISPR/Cas9 target for *pksA* gene in *P. roqueforti* |
| Pro_pks1P13f | TTCCAGGGGACAGCTTCAGATG | Diagnostic PCR to confirm mutation on *pksA* locus in *P. roqueforti* |
| Pro_pks1P14r | TCACCTCGGTTCAGCAAAGTCA | Diagnostic PCR to confirm mutation on *pksA* locus in *P. roqueforti* |
| Pro_ku70P11f | CTGCTCGGTTAATCTTACTAGACGAGCTTACTCGTTTCG | Creating CRISPR/Cas9 target for *kusA* gene in *P. roqueforti* |
| Pro_ku70P12r | TAGTAAGATTAACCGAGCAGGTTTTAGAGCTAGAAATAGCAAG | Creating CRISPR/Cas9 target for *kusA* gene in *P. roqueforti* |
| Pro_ku70P15f | TGCCTCACCGGTCTTAGCTGCT | Diagnostic PCR *kusA* mutation in *P. roqueforti* |
| Pro_ku70P16r | GCCTTGGGAAGCTGCAATTGGC | Diagnostic PCR *kusA* mutation in *P. roqueforti* |
| Pva_pksAP11r | CCGATCAATGTCGAGAAGCCGACGAGCTTACTCGTTTCG | Creating CRISPR/Cas9 target for  *pvpP* gene in *P. variotii* |
| Pva_pksAP12f | GGCTTCTCGACATTGATCGGGTTTTAGAGCTAGAAATAGCAAG | Creating CRISPR/Cas9 target for  *pvpP* gene in *P. variotii* |
| Pva_ku70P11f | CGTCTAATGCGAGAGCCTGCGACGAGCTTACTCGTTTCG | Creating CRISPR/Cas9 target for  *kusA* gene in *P. variotii* |
| Pva_ku70P12r | GCAGGCTCTCGCATTAGACGGTTTTAGAGCTAGAAATAGCAAG | Creating CRISPR/Cas9 target for  *kusA* gene in *P. variotii* |
| Pva_pksAP17f | ACATTCTCTTGGGCACGGAGAA | Diagnostic PCR to confirm mutation on  *pvpP* locus in *P. variotii* |
| Pva_pksAP18r | ACGCTTGGTCCTGCTGACTTTA | Diagnostic PCR to confirm mutation on  *pvpP* locus in *P. variotii* |
| Pva_pksAP19f | AAGCCTCTGATCGCCAAGAACT | Diagnostic PCR to check presence of *pvpP* |
| Pva_pksAP20r | AAGGTCCTTGACAGTCGGATGG | Diagnostic PCR to check presence of *pvpP* |
| Pva_pksAP22f | TCTCCGATCAACTGCGGGCAGA | Diagnostic PCR outside of flanks to see ‘clean’ knock-out of *pvpP* |
| Pva_pksAP23r | AACTTGTTCGAGCACGCGAGGG | Diagnostic PCR outside of flanks to see ‘clean’ knock-out of *pvpP* |
| Pva_ku70P15f | TCGCTTCTCAGCTTTGCAATGG | Diagnostic PCR *kusA* mutation in *P. variotii* |
| Pva_ku70P16r | TTCGATTTTCCGGTACTGGGCT | Diagnostic PCR *kusA* mutation in *P. variotii* |
