# Supplementary material for: Preservation stress resistance of melanin deficient conidia from Paecilomyces variotii and Penicillium roqueforti mutants generated via CRISPR/Cas9 genome editing
Source: Fungal Biol Biotechnol. 2021 Apr 2;8:4. doi: 10.1186/s40694-021-00111-w (PMC8017634; doi:10.1186/s40694-021-00111-w)
Supplement: Supplementary file 7 — Additional file 7. Protocol S1. A detailed protocol for CRISPR/Cas9 mediated gene knock-out in Penicillium roqueforti and Paecilomyces variotii. [file 40694_2021_111_MOESM7_ESM.pdf]

Target design

1. Go to http://chopchop.cbu.uib.no/
2. Paste the fasta sequence of your gene of interest (GOI) and select the correct species ("*Penicillium roqueforti* (FM164)" or "*Paecilomyces variotii* (CBS101075)")
3. Click "Find Target Sites!" and select a suitable target (ideally self-complementarity = 0 and there are no off-targets).
4. Locally blast the target to the strain(s) you want to transform to make sure that there are no SNPs or off-target effects.
5. Design primers according to the table below. Only paste the 20 bp target. Do not include the 3 bp PAM site.

#	Primer	Sequence (5' --> 3')	Remark
1	pTE1_for	CCttaattaaACTCCGCCGAACGTACTG	Forward primer on promoter. Contains PacI site for ligation (lower case)
2	gRNA_GOI_Rv	xxxxxxxxxxxxxxxxxxxxGACGAGCTTACTCGTTTCG	Replace "xxx...xxx" with your **(R&C)** target sequence. Primes at the 3' end of the promoter.
3	gRNA_GOI_Fw	xxxxxxxxxxxxxxxxxxxxGTTTTAGAGCTAGAAATAGCAAG	Replace "xxx...xxx" with your target sequence. Primes at the 5' end of the crRNA sequence.
4	pTE1_rev	CCttaattaaAAAAGCAAAAAAGGAAGGTACAAAAAAGC	Reverse primer on terminator. Contains PacI site for ligation (lower case)

Guide RNA construction

1. Pipette the PCR mixtures according to the table below:

Ingredients	Volume (µL)
MQ	28.5
Buffer (5x) HF	10
dNTPs mix (1.25mM each)	8
Forward primer (10 µM)*	1
Reverse primer (10 µM)*	1
DNA (pTLL108.1 or pTLL109.2)**	1 (~2ng)
Phusion polymerase	0.5
**Total**	**50**

* Use primers 1 and 2 for the 5' flank. Use primers 3 and 4 for the 3' flank.
** Use pTLL108.1 for the 5' flank. Use pTLL109.2 for the 3' flank.

2. Perform PCR using the following programs:

5' flank

Cycles	Step	Temp (°C)	Time
1	Initial denaturation	98	30"
30	Denaturation	98	5"
	Annealing	60	5"
	Extension	72	**6"**
1	Final Extension	72	30"
	Hold	4	

3' flank

Cycles	Step	Temp (°C)	Time
1	Initial denaturation	98	30"
30	Denaturation	98	5"
	Annealing	60	5"
	Extension	72	**2"**
1	Final Extension	72	30"
	Hold	4	

3. Run 5 µL of the PCR products on a 1% agarose gel at 120V for 40 minutes. 5’ and 3’ fragments should be 291 and 131 basepairs (bp) respectively.
4. Purify the fragments from the PCR mix using the thermoscientific GeneJET Gel Extraction Kit. Only isolate the fragments from gel when secondary bands are showing. To do this load the complete PCR mixture on gel and cut out the correct bands.
5. Measure the DNA concentration using a nanodrop.
6. Pipette 5 PCR mixtures according to the table below:

Ingredients	Volume (µL)
MQ	27.5
Buffer (5x) HF	10
dNTPs mix (1.25mM each)	8
Forward primer pTE1_for (10 µM)	1
Reverse primer pTE1_ rev (10 µM)	1
5’ fragment (2 ng/µL)	1
3’ fragment (2 ng/µL)	1
Phusion polymerase	0.5
**Total**	**50**

7. Perform PCR using the following program:

5' flank + 3' flank fusion

Cycles	Step	Temp (°C)	Time
1	Initial denaturation	98	30"
30	Denaturation	98	5"
	Annealing	60	5"
	Extension	72	**12"**
1	Final Extension	72	2'
	Hold	4	

8. Run 5 µL of the PCR product on a 1% agarose gel at 120V for 40 minutes. The fragment should be 402 bp.
9. Purify the fragment from all 5 PCR mixtures over 1 column using the thermoscientific GeneJET Gel Extraction Kit. Only isolate the fragment from gel when secondary bands are showing. To do this load the complete PCR mixture on gel and cut out the correct bands.
10. Measure the DNA concentration using a nanodrop.
11. Store at -20°C.

Guide RNA cloning in pJET

Alternatively, the PCR product can be digested overnight (1h has also worked) with PacI. This can then be directly ligated into pFC332 and would save time.

Ligation

1. Add to a 1.5 mL tube:
   - 14 µL guide RNA (gRNA) construct
   - 4 µL 5x fast ligase buffer
   - 1 µL pJET
   - 1 µL T4 DNA ligase

   Mix and centrifuge shortly
2. Incubate for 5 minutes at room temperature (RT).
3. Store at -20°C.

E. coli transformation

4. Add 10 µL ligation mixture to 200 µL competent *E. coli* cells and gently mix. Include controls when desired.
5. Incubate for 30 minutes on ice.
6. Heatshock for 45 seconds at 42°C and 1000 rpm, or for 60 seconds without shaking.
7. Incubate for a minimum of 2 minutes on ice.
8. Add 900 µL LB and mix.
9. Incubate for 45 minutes at 37°C and 1000 rpm, or for 60 minutes without shaking.
10. Plate 100 µL on a LB agar plate with 100 µg/mL ampicillin (LB+amp plate).
11. Dilute 10 µL in 90 µL LB and plate on a LB+amp plate.
12. Incubate plates overnight (O/N) at 37°C.

Plasmid isolation

13. Pick 2 colonies using a sterile toothpick and inoculate 2.5 mL LB+amp in a 13 mL tube.
14. Incubate tubes overnight at 37°C and 250 rpm.
15. Add 500 µL culture to 500 µL 97% glycerol and store at -80°C.
16. Centrifuge the remaining 2 mL for 10 minutes at 3500 rpm and remove supernatant.
17. Isolate plasmid DNA using the thermoscientific GeneJET Plasmid Miniprep Kit.
18. Measure the DNA concentration using a nanodrop.

PacI restriction of pJET

19. Add to a 1.5 mL tube:
    - 17 µL plasmid
    - 2 µL Buffer PacI
    - 1 µL PacI
20. Mix and centrifuge shortly.
21. Incubate at 37°C for 1 hour.
22. Deactivate at 65°C for 20 minutes.
23. Load the complete mixture on a 1% agarose gel and run at 120V for 40 minutes.
24. Cut the gRNA fragment (402 bp) from the gel and purify the DNA using the thermoscientific GeneJET Gel Extraction Kit.
25. Measure the DNA concentration using a nanodrop.
26. Send all plasmids that showed correct bands (2974 bp and 402 bp) for sequencing.

Vector construction

! Perform the following steps (PacI restriction of pFC332 – *E. coli* transformation) directly after each other while keeping the plasmid on ice as much as possible. !

PacI restriction of pFC332

1. Add to a 1.5 mL tube:
   - 17 µL pFC332
   - 2 µL Buffer PacI
   - 1 µL PacI
2. Mix and centrifuge shortly.
3. Incubate at 37°C for 1 hour.
4. Deactivate at 65°C for 20 minutes.
5. Load the complete mixture on a 1% agarose gel and run at 120V for 40 minutes.
6. Cut the gRNA linearized plasmid (15561 bp) from the gel and purify the DNA using the thermoscientific GeneJET Gel Extraction Kit.

Defosforylation

7. Add to a 1.5 mL tube:
   - 17 µL linearized pFC332 (cut with PacI)
   - 2 µL FastAP buffer
   - 1 µL FastAP
8. Mix and centrifuge shortly.
9. Incubate for 10 minutes at 37°C.
10. Incubate for 5 minutes at 75°C.

Ligation

11. Add to a 1.5 mL tube:
    - 12 µL gRNA (cut from pJET with PacI, or digested O/N with PacI)
    - 3 µL linearized and defosforylated pFC332
    - 4 µL fast ligase buffer
    - 1 µL T4 DNA ligase
12. Incubate for 1 hour at RT.

E. coli transformation

13. Add 10 µL ligation mixture to 200 µL competent *E. coli* cells and gently mix. Include controls when desired.
14. Incubate for 30 minutes on ice.
15. Heatshock for 45 seconds at 42°C and 1000 rpm, or for 60 seconds without shaking.
16. Incubate for a minimum of 2 minutes on ice.
17. Add 900 µL LB and mix.
18. Incubate for 45 minutes at 37°C and 1000 rpm, or for 60 minutes without shaking.
19. Plate 100 µL on a LB+amp plate.
20. Centrifuge the remaining 1 mL for 5 minutes at 6000 rpm.
21. Remove the supernatant and resuspend the pellet in 100 µL LB.
22. Plate on a LB+amp plate.
23. Incubate plates overnight (O/N) at 37°C.

Plasmid isolation

1. Pick 3 or more colonies using a sterile toothpick and inoculate 2.5 mL LB+amp in a 13 mL tube.
2. Incubate tubes overnight at 37°C and 250 rpm.
3. Add 500 µL culture to 500 µL 97% glycerol and store at -80°C.
4. Centrifuge the remaining 2 mL for 10 minutes at 3500 rpm and remove supernatant.
5. Isolate plasmid DNA using the thermoscientific GeneJET Plasmid Miniprep Kit.
6. Measure the DNA concentration using a nanodrop.

SacII restriction of vectors

1. Add to a 1.5 mL tube:
   - 14 µL sterile water
   - 3 µL plasmid (200-700 ng/µL)
   - 2 µL buffer B
   - 1 µL SacII (Cfr42I)
2. Incubate for 1 hour at 37°C.
3. Deactivate for 20 minutes at 65°C.
4. Load 5 µL on a 1% agarose gel and run at 120V for 40 minutes. Bands of 14381bp, 1077 bp and 497 bp should be visible.

***P. roqueforti/P. variotii* Transformation**

Protoplastation

1. Inoculate 500 µL spore solution in 100 mL CM in a 300 mL shaking flask.
2. *P. roqueforti*: Incubate for 41 hours at 25°C and 200 rpm.
   P. variotii: Incubate for 17 hours at 30°C and 200 rpm.
3. Dissolve 200 mg lysing enzymes (Lysing Enzymes from Trichoderma harzianum, Sigma) in 10 mL SMC. Carefully dissolve, then filter sterilize the solution over a 20 µm filter.
4. Collect mycelium from the overnight culture on a myracloth filter. Wash on the filter with SMC.
5. Add to a maximum of 0.5 g mycelium (wet weight)* to the protoplastation solution.
   * *I never weigh the mycelium, but simply add about three scoops of mycelium with a sterile cotton stick.*
6. Horizontally shake the solution at 37°C and 80 rpm.
7. Check for protoplasts under the microscope after 15 minutes and then every 15 minutes.
8. Gently mix by pipetting and collect the protoplasts through a myracloth filter in a 50 mL falcon tube. Wash with STC.
9. Centrifuge for 10 minutes at 2000 xg and 10°C.
10. Meanwhile, make a PEG solution by dissolving 0.25 g/mL in TC. You will need 1.025 mL per transformation.
11. Carefully discard the supernatant and resuspend the pellet in 1 mL STC. Transfer to a 1.5 mL tube.
12. Centrifuge for 5 minutes at 3000 xg and discard the supernatant.
13. Resuspend the pellet in 1 mL STC.
14. Centrifuge for 5 minutes at 3000 xg and discard the supernatant.
15. Resuspend the pellet in 100 µL STC per transformation. Keep on ice.

Transformation

16. Add 2µg plasmid DNA, 100µL protoplasts and 25 µL freshly made PEG solution to a 50 mL tube.*
    * Add ~2µg donor DNA when required
17. Add 1 mL freshly made PEG solution and mix.
18. After exactly 5 minutes, add 2 mL STC and mix.
19. Add 20 mL top agar (containing 200 µg/mL hygromycin (hyg)), mix and pour onto a 50 mL MMS agar plate (containing 200 µg/mL hyg)*
    * Some strains may need different concentrations of hyg. Use roughly 5-10 times the MIC value.
20. *P. roqueforti*: Incubate at 25°C until sporulating colonies appear (~6-8 days).
    P. variotii: Incubate at 30°C until sporulating colonies appear (~6-8 days).

Purification

21. Single streak spores from single colonies on MM agar plates containing 100 µg/mL hyg.
22. *P. roqueforti*: Incubate at 25°C until sporulating colonies appear (~5 days)
    P. variotii: Incubate at 30°C until sporulating colonies appear (~3 days)
23. Single streak spores from single colonies on MM agar plates.
24. *P. roqueforti*: Incubate at 25°C until sporulating colonies appear (~5 days)
    P. variotii: Incubate at 30°C until sporulating colonies appear (~3 days)

25. Spot spores from single colonies on MM agar plates containing 100 µg/mL hyg, and streak them (using the same sterile cotton stick) on malt extract agar (MEA) plates to make spore plates.
26. *P. roqueforti*: Incubate at 25°C until sporulating colonies appear (~5 days)
    P. variotii: Incubate at 30°C until sporulating colonies appear (~3 days)
27. Transformants growing on MEA, but not on MM+hyg, have successfully lost the vector. Use spores from the MEA plates for analysis.

Analysis

Phenol free DNA isolation (adapted from Paege, 2017)

P. variotii:

1. Inoculate 1-3 ml CM medium with spores from the MEA plate in a 15 ml tube using a cotton stick and incubate overnight horizontally in a rack at 30°C.
2. Harvest the mycelium of each strain into a fresh 2 ml Eppendorf tube using the back end of a cotton stick and seal the tube with an additional cap from another 2ml tube with small holes.
3. Freeze samples in liquid nitrogen or for 30 minutes at −80°C.
4. Dry the samples overnight in a freeze-dryer.
5. Grind the mycelium using cotton sticks (not sterile) and make sure you take less than 0,5 ml volume for further extraction.

P. roqueforti:

1. Inoculate 20 mL CM medium with spores from the MEA plate in a 100 mL shaking flask.
2. Incubate the flask for 2 days at 25°C and 200 rpm.
3. Dry the mycelium on a filter connected to a vacuum pump.
4. Pour liquid nitrogen into a mortar and add the dried mycelium.
5. Grind the frozen mycelium using pestle and mortar.

Both:

6. Resuspend the pulverized cells in 500 µl of extraction buffer.
7. Heat for 15min at 65°C while shaking at 1000 rpm, or vortex occasionally.
8. Cool down on ice for 5min.
9. Add 100 µl 8M potassium acetate , invert 8-10 times.
10. Centrifuge at 13000 rpm for 15 minutes.
11. Transfer the supernatant to a new 1.5 ml tube.
12. Optional: redo from step 9 if the supernatant looks yellowish.
13. Add 300 µl isopropanol and mix.
14. Centrifuge at 13000 rpm for 15 minutes.
15. Wash pellet with 1 ml 70% EtOH (without resuspending).
16. Centrifuge at 13000 rpm for 2 minutes.
17. Discard supernatant and dry pellet at 42°C (up to 65°C).
18. Resuspend the DNA in 50 - 100 µl of H2O containing 2 µl 10 mg/ml RNAse A.
19. incubate the solution at 65°C for 30 min while shaking at 1000 rpm (or vortex occasionally) to completely dissolve the DNA and degrade the RNA.
20. Load 2-3 µl DNA on a 1% agarose gel to check the quality of your purification. The gDNA should appear as one band above 10 kbp.
21. Measure the concentration using a Nanodrop.

Analysis

22. Design primers 200 bp up- and downstream of the Cas9 cutting site.
23. Pipette the PCR mixture according to the table below and perform PCR:

Ingredients	Volume (µL)
MQ	28.5
Buffer (5x) HF	10
dNTPs mix (1.25mM each)	8
Forward primer (10 µM)	1
Reverse primer (10 µM)	1
gDNA	1
Polymerase	0.5
**Total**	**50**

24. Run 5 µL of the PCR product on a 1% agarose gel at 120V for 45 minutes.
25. When correct bands are showing, purify the fragment from the PCR mixture using the thermoscientific GeneJET Gel Extraction Kit.
26. Measure the DNA concentration using a nanodrop.
27. Send the fragments for sequencing to confirm an insertion, deletion or mutation.

Materials (from Arentshorst et. al., 2012):

1. Glucose (50%): For 1 L: Boil 500 mL Milli-Q (MQ) in a 1,000 mL beaker on a heated magnetic stirrer. Slowly add 500 g of D (+)-glucose anhydrous. After glucose has been dissolved, let the solution cool down to RT, add MQ up to 1 L and autoclave.

2. ASPA + N (50×): For 1 L: Add 297.5 g $NaNO_3$, 26.1 g KCl and 74.8 g KH_2PO_4 to 600 mL MQ in a 1 L cylinder. When all salts are dissolved, set pH to 5.5 with KOH (use 5 M KOH). Add MQ up to 1 L and autoclave.

4. $MgSO_4$ (1 M): For 1 L: Add 246.5 g $MgSO_4 \cdot 7H_2O$ to 600 mL MQ in a 1 L cylinder. When all salts are dissolved, add MQ up to 1 L and autoclave.

5. Trace element solution (1,000×): For 1 L: Add 10 g EDTA, 4.4 g $ZnSO_4 \cdot 7H_2O$, 1.01 g $MnCl_2 \cdot 4\ H_2O$, 0.32 g $CoCl_2 \cdot 6\ H_2O$, 0.315 g $CuSO_4 \cdot 5\ H_2O$, 0.22 g $(NH_4)_6Mo_7O_{24} \cdot 4H_2O$, 1.11 g $CaCl_2$ and 1.0 g $FeSO_4 \cdot 7H_2O$ to 600 mL MQ. When dissolved, set pH to 4.0 with 1 M NaOH and 1 M HCl, fill MQ up to 1 L and autoclave (see Note 1).

13. Hygromycin (100 mg/mL): Dissolve 1 g of hygromycin in 10 mL of MQ, sterilize by filtration, make aliquots of 500 m L and store at −20°C. The final concentration in the medium is 100 m g/mL, except for transformation plates, then use 200 mg/mL (see Note 6).

14. Caffeine (50 mg/mL): For 100 mL: Add 5 g of caffeine to 50 mL of warm MQ (about 50–60°C) in a 100 mL cylinder. When caffeine is dissolved, add MQ up to 100 mL and sterilize by filtration. The final concentration in cultivation medium is 500 mg/mL.

15. Minimal medium (MM): For 500 mL: Add under sterile conditions to 480 mL of sterile MQ: 10 mL of 50% glucose , 10 mL of 50 × ASPA + N, 1 mL of 1 M MgSO 4 and 500 m L of 1,000× trace element solution. For MM + agar, autoclave 480 mL of MQ with 7.5 g of agar (Scharlau) and add all components after autoclaving under sterile conditions (see Note 7).*
* *I always add all components before autoclaving.*

19. Minimal medium + sucrose + agar (MMS): For 500 mL: Dissolve 162.6 g of D (+)-saccharose in 480 mL of MQ, add 6 g (1.2%) of agar and autoclave (see Note 9). For hygromycin selection, add after autoclaving under sterile conditions: 10 mL of 50× ASPA + N, 1 mL of 1 M $MgSO_4$, 500 mL of 1,000× trace element solution, 5 mL of 50 mg/mL caffeine and 1 mL of 100 mg/mL hygromycin (see Note 10).*
* *I always add all components except caffeine and hygromycin before autoclaving.*

20. MMS top agar: For 500 mL: Dissolve 162.6 g of D (+)-saccharose in 480 mL of MQ, add 3 g of agar (final concentration is 0.6%) and autoclave (see Note 9). Store the top agar at 65°C and add under sterile conditions: 10 mL of 50× ASPA + N, 1 mL of 1 M $MgSO_4$, 500 m L of 1,000× trace element solution. Before use, transfer the top agar to a 47–50°C water bath.*
* *I also add caffeine and hygromycin in the same concentrations as in MMS.*

21. Complete medium (CM): For 500 mL: Add 0.5 g casamino acids, 2.5 g yeast extract and if required, 7.5 g agar to 480 mL of MQ and autoclave. Afterwards, add under sterile conditions: 10 mL of 50% glucose, 10 mL of 50× ASPA + N, 1 mL of 1 M $MgSO_4$, 500 m L of 1,000× trace element solution.

22. SMC: For 1 L: Add 242.3 g D -sorbitol, 5.5 g $CaCl_2$ and 3.9 g MES hydrate to 600 mL MQ in a 1 L cylinder. When everything is dissolved, set pH to 5.8 using 1 M NaOH and 1 M HCl. Add MQ up to 1 L and autoclave.

23. TC: For 1 L: Add 5.5 g $CaCl_2$ and 1.2 g Tris to 800 mL MQ in a 1 L cylinder. When everything is dissolved, set pH to 5.8 using 1 M NaOH and 1 M HCl. Add MQ up to 1 L and autoclave.

24. STC: For 1 liter: Add 242.3 g D -sorbitol to 600 mL TC in a 1 L cylinder. When sorbitol is dissolved, add TC up to 1 L and autoclave.

25. PEG buffer: For 10 mL: Add 2.5 g of Polyethylene glycol 6000 (PEG) to a 50-mL tube, add TC up to 10 mL under sterile conditions and dissolve PEG by shaking. Use PEG solution only fresh.

Notes:

1. The color of the 1,000× trace element solution is green when freshly made. After autoclaving, the color changes from green to purple within 2 weeks.

7. For all selective media, the type and brand of agar needs to be tested. Agar might contain impurities which can inhibit growth or, alternatively, can cause background growth on selective medium (trace amounts of nitrogen are sufficient to allow background growth of an amdS$^-$ strain on MM-AA agar).

9. The addition of caffeine to hygromycin selection plates strongly reduces background growth and thereby improves selectivity (C. de Bekker and H. Wösten, personal communication).

Materials (adapted from Paege, 2017)

1. DNA extraction buffer: For 250 mL: Dissolve 1.25 g SDS, 0.61 g Tris, 0.18 g EDTA and 0.37 g NaCl in 200 mL MQ. When everything is dissolved, set pH to 8.0 using 1 M NaOH and 1 M HCl. Add MQ to 250 mL and autoclave.

2. 8M Potassium acetate: For 50 mL: Dissolve 39.26 g potassium acetate in 50 mL MQ and autoclave.

References:

- Arentshorst, M., Ram, A. F. J., & Meyer, V. (2012). Using Non-homologous End-Joining-Deficient Strains for Functional Gene Analyses in Filamentous Fungi. In *Methods in Molecular Biology* (pp. 133–150). https://doi.org/10.1007/978-1-61779-501-5_9
- Paege, N. (2017) SOP017: Phenol free DNA Extraction, Applied and Molecular Microbiology, TU Berlin.
